# Bioactive Compounds from *Cucurbita* spp. and Their Processing By-Products: A Critical Review

**DOI:** 10.3390/metabo16100754

**Published:** 2026-10-08

**Authors:** Bela Kolarš, Ivana Minaković, Milana Bosanac, Nemanja Gvozdenović, Jovanka Milak, Nemanja Petrović, Vesna Mijatović Jovin

**Affiliations:** 1 Department of General Medicine and Geriatrics, Faculty of Medicine, University of Novi Sad, 21000 Novi Sad, Serbia; bela.kolars@mf.uns.ac.rs; 2 Health Center “Novi Sad”, 21000 Novi Sad, Serbia; 3 Department of Histology and Embryology, Faculty of Medicine, University of Novi Sad, 21000 Novi Sad, Serbia; milana.bosanac@mf.uns.ac.rs; 4 Department of Emergency Medicine, Faculty of Medicine, University of Novi Sad, 21000 Novi Sad, Serbia; nemanja.gvozdenovic@mf.uns.ac.rs; 5 Clinic for Orthopedic Surgery and Traumatology, University Clinical Center of Vojvodina, 21000 Novi Sad, Serbia; 6 Clinic of Nephrology and Clinical Immunology, University Clinical Center of Vojvodina, 21000 Novi Sad, Serbia; 910104d25@mf.uns.ac.rs; 7 Doctoral Academic Studies, Faculty of Medicine, University of Novi Sad, 21000 Novi Sad, Serbia; dr.nemanja.petrovic@gmail.com; 8 Clinic of Cardiovascular Surgery, Institute for Cardiovascular Diseases of Vojvodina, 21208 Sremska Kamenica, Serbia; 9 Department of Pharmacology, Toxicology and Clinical Pharmacology, Faculty of Medicine, University of Novi Sad, 21000 Novi Sad, Serbia; vesna.mijatovic-jovin@mf.uns.ac.rs

**Keywords:** *Cucurbita*, pumpkin by-products, phytochemical profiling, green extraction, processing, bioaccessibility, biological activity, circular bioeconomy

## Abstract

**Highlights:**

**What are the main findings?**

- Pumpkin tissues and processing by-products are complementary sources of carotenoids, phenolics, proteins, unsaturated lipids, phytosterols, polysaccharides, and dietary fiber.
- Processing can improve phytochemical recovery or simulated bioaccessibility, while evidence for health benefits remains largely preclinical and preparation-specific.

**What are the implications of the main findings?**

- Reliable comparisons require authenticated plant materials, validated analytical profiles, and standardized preparations.
- Pumpkin by-products offer near-term opportunities as food ingredients; health-related claims require well-designed human trials.

**Abstract:**

Pumpkin (*Cucurbita* spp.) processing generates peels, seeds, seed coats, pomace, and press cakes containing carotenoids, phenolics, proteins, unsaturated lipids, tocopherols, phytosterols, polysaccharides, and dietary fiber. This critical narrative review integrates composition and analytical characterization across anatomical fractions with extraction technologies, processing effects, biological evidence, and application readiness. Conventional extraction, pressing, supercritical carbon dioxide, deep eutectic solvents, enzyme-assisted extraction, ultrasound, microwaves, pulsed electric fields, and high-pressure homogenization differ in selectivity, recovery, and scalability. The reviewed evidence does not establish the environmental superiority of green extraction technologies without full process-level assessment. Processing can improve extractability or simulated bioaccessibility but also cause degradation or losses; concentration following water removal does not establish true retention. Experimental studies support potential antioxidant, anti-inflammatory, glycemia-related, antimicrobial, and antiproliferative activities, but most evidence remains chemical, cellular, or animal-based. Human evidence is preparation-specific and most developed for lower urinary tract symptoms; controlled evidence for glycemic and other systemic outcomes remains limited or inconsistent. Near-term opportunities include food ingredients, oils, protein fractions, pigment delivery, and selected packaging materials. Translation requires authenticated materials, validated analytical fingerprints, standardized preparations, safety and stability testing, adequately powered clinical trials for health-related claims, and mass, energy, economic, and life-cycle assessments.

## 1. Introduction

The genus *Cucurbita*, native to the New World, comprises trailing or climbing herbaceous plants and belongs to the Cucurbitaceae, a large and economically important family comprising approximately 95 genera and 950–1000 species [1]. The genus includes both annual and perennial taxa, and estimates of species number vary according to the taxonomic authority and treatment of species boundaries. Five domesticated species are recognized within *Cucurbita*: *C. argyrosperma*, *C. ficifolia*, *C. maxima*, *C. moschata*, and *C. pepo*. These species have distinct domestication histories in the Americas; evidence supports multiple independent domestication events within the genus, but the archaeological and genetic evidence is not equally complete for all taxa [2]. Archaeological material from Coxcatlan Cave documents domesticated *C. pepo* by approximately 7900 calibrated years before present [3]. Among these species, *C. pepo*, *C. maxima*, and *C. moschata* dominate international commercial production, whereas *C. argyrosperma* and *C. ficifolia* retain greater regional importance. Because the common terms pumpkin, squash, and gourd overlap across regions and cultivar groups, this review uses “pumpkin” broadly for edible cultivated *Cucurbita* while identifying the species whenever the underlying evidence is species-specific.

According to FAOSTAT’s (Food and Agriculture Organization Corporate Statistical) Crops and livestock products dataset (QCL), global production in the category “Pumpkins, squash and gourds” (item code 394) reached approximately 34.6 million metric tons from a harvested area of 2.25 million hectares in 2024 [4]. The category is broader than pumpkin alone and is not resolved by *Cucurbita* species, but it demonstrates the agricultural and industrial scale of the commodity group. Pumpkins are consumed fresh and processed into purées, juices, soups, bakery ingredients, snacks, seed oils, and other formulated foods. Processing generates peels or rinds, seeds, seed coats, fibrous pomace, and press cakes that are often treated as low-value residues despite retaining proteins, unsaturated lipids, carotenoids, tocopherols, phytosterols, phenolic compounds, and structural polysaccharides. Growing emphasis on waste prevention and the circular bioeconomy, which seeks to keep renewable biological resources in productive use through recovery and cascading applications, has stimulated interest in pumpkin-residue valorization. Here, valorization refers to converting low-value fractions into higher-value food ingredients, nutraceutical candidates, feed materials, active packaging components, and other products [5].

*Cucurbita* species also have a long history of use in traditional food and medical practices. Pumpkin seeds have been employed as vermifuges or anthelmintic preparations in several traditional systems, *C. ficifolia* fruit has been used in Mexican traditional medicine for glycemic control, and *C. pepo* seed preparations have a documented history of use in Central and Southeastern Europe for lower urinary tract symptoms (LUTS) associated with benign prostatic enlargement [6,7,8]. These practices provided hypotheses for subsequent pharmacological and clinical investigation, but historical use is not equivalent to demonstration of efficacy. Traditional preparations may also differ substantially from contemporary oils, solvent extracts, purified fractions, or multi-ingredient supplements in composition, dose, and exposure.

The scientific interest in pumpkin-derived materials stems from the complementary composition of their anatomical fractions. Pulp is principally valued for carotenoids, carbohydrates, and structural polysaccharides; seeds and seed oil provide proteins, unsaturated fatty acids, tocopherols, phytosterols, and phenolic compounds; and peels, rinds, pomace, seed coats, and press cakes retain pigments, fiber, residual lipids, proteins, and other extractable constituents [9]. Flowers, leaves, and germinating tissues may provide additional specialized metabolites and bioactive proteins, although these materials are less extensively characterized. Pumpkin-derived compounds and preparations have demonstrated antioxidant, anti-inflammatory, antimicrobial, glycemia-related, immunomodulatory, neurobehavioral, and antiproliferative activities, predominantly in chemical assays, cell systems, and animal models [6]. Human evidence remains comparatively limited and preparation-specific. It is most developed for selected pumpkin seed preparations evaluated for LUTS, whereas controlled evidence for glycemic and other systemic outcomes remains limited or inconsistent [10,11]. Pumpkin tissues and extracts should therefore be regarded as promising sources of functional ingredients and standardized nutraceutical candidates rather than established agents for preventing or treating multiple diseases.

The composition and potential functionality of pumpkin-derived products are strongly influenced by postharvest handling, storage, and processing [12]. Mechanical disruption, blanching, cooking, roasting, drying, freezing, fermentation, and pressure-assisted treatments may alter chemical stability, tissue structure, release from the food matrix, sensory quality, and shelf life [9]. These effects are not uniformly beneficial or detrimental: disruption and controlled heating may increase the extractability or in vitro bioaccessibility of some compounds, whereas prolonged exposure to heat, oxygen, or light may promote oxidation, isomerization, or loss of thermolabile constituents [12]. Extractability, simulated bioaccessibility, and human bioavailability should therefore be distinguished. Extractability refers to the fraction recovered under a specified analytical or processing procedure; simulated bioaccessibility denotes the fraction released from the food matrix during in vitro digestion and potentially available for absorption, whereas bioavailability additionally requires absorption and systemic distribution or utilization in vivo. Emerging extraction and intensification methods—including supercritical carbon dioxide (SC–CO_2_), enzyme-assisted aqueous extraction (EAAE), deep eutectic solvents (DES), ultrasound, microwaves, pulsed electric fields (PEFs), and high-pressure homogenization (HPH)—may reduce treatment time, modify selectivity, or limit the use of volatile petrochemical solvents [13]. However, no technology is universally superior, and claims of reduced environmental impact require supporting data on solvent recovery, mass and energy balances, water use, waste generation, process economics, and life-cycle performance.

Despite rapidly increasing publication activity, the evidence base remains difficult to integrate. Composition varies with species, cultivar, anatomical fraction, maturity, cultivation environment, storage, and analytical method, while extraction and processing studies frequently omit parameters required for replication. Results are also reported inconsistently on fresh-, wet-, or dry-mass bases and do not always distinguish concentration caused by water loss from true compound retention. In biological research, whole foods, seed oils, crude extracts, purified polysaccharides, isolated compounds, and multi-ingredient products are sometimes discussed as though they were equivalent interventions. This heterogeneity limits cross-study comparison and complicates attribution of biological effects to defined exposures. Additional gaps include limited extract standardization, insufficient pharmacokinetic and human bioavailability data, few adequately powered clinical trials, incomplete assessment of concentrated-extract safety, and a shortage of pilot-scale, sensory, techno-economic, and life-cycle evaluations. To ensure consistent terminology, this review uses functional food to denote a conventional food or food formulation intended to provide benefits beyond basic nutrition, whereas nutraceutical denotes a concentrated food-derived preparation, such as a standardized extract or capsule. These are operational definitions rather than universally harmonized regulatory categories.

Recent reviews have summarized the composition and potential uses of pumpkin and its derivatives. Gavril et al. reviewed phytochemicals, extraction methods, biological activities, and food and industrial applications of pumpkin and its by-products, while Batool et al. emphasized nutritional composition, phytochemical constituents, and reported therapeutic potential [5,14]. Building on these contributions, the present review places particular emphasis on the comparability of study findings and the evidence required to translate them into practical applications. Its added contribution is an integrated appraisal linking the chemical characterization and standardization of individual *Cucurbita* fractions with processing effects, the strength of biological evidence, and technological readiness. Specifically, it distinguishes changes in measured concentration from true compound retention and separates analytical extractability, in vitro bioaccessibility, and human bioavailability. Biological findings are evaluated according to experimental level, from chemical and enzyme assays to cell studies, animal experiments, and human trials, with attention to the species, fraction, and preparation actually tested. Extraction technologies and product applications are further assessed in relation to demonstrated performance, safety, scalability, and the economic and environmental data needed to substantiate their feasibility. This approach identifies the analytical requirements, limitations of biological evidence, and development priorities relevant to the sustainable conversion of pumpkin by-products into reproducible food ingredients, nutraceutical candidates, and functional materials.

## 2. Review Methodology

This article was prepared as a critical narrative review rather than a systematic review or meta-analysis. Literature was identified through structured, iterative searches of PubMed, Crossref-indexed records, and publisher databases, supplemented by FAOSTAT and relevant European regulatory documents. The initial literature search covered publications available online by 20 July 2026. Targeted source checks and updates to statistical, regulatory, and bibliographic information were undertaken during manuscript revision; access dates for online resources are reported in the reference list. Search concepts combined *Cucurbita* or pumpkin with terms relating to anatomical fractions and by-products; carotenoids, phenolics, seed oil, proteins, peptides, polysaccharides, metabolomics, and phytochemical profiling; SC–CO_2_, DES, enzyme-assisted extraction, ultrasound, microwaves, PEF, and high-pressure processing; cooking, drying, roasting, fermentation, storage, bioaccessibility, and stability; and cell, animal, clinical, functional-food, nutraceutical, packaging, and cosmetic applications. Reference lists of relevant primary studies and recent reviews were also screened to identify additional publications.

Peer-reviewed primary research was prioritized for quantitative composition, extraction performance, processing effects, mechanisms, and human outcomes. Recent reviews were used to map the field and locate source studies. Agricultural production statistics were checked against FAOSTAT data [4], while regulatory statements were checked against European Medicines Agency documents [7], relevant European Union legislation, and European Food Safety Authority guidance [15,16,17].

Studies were retained when the *Cucurbita* species or matrix, intervention or process, and measured outcome could be identified. Evidence was organized by anatomical fraction and by level: chemical or enzyme assay, cultured cell, animal model, uncontrolled human study, controlled trial, or pooled clinical analysis. Whole foods, oils, crude extracts, purified fractions, and multi-ingredient formulations were treated as distinct exposures.

Interpretation emphasized analytical basis, comparator choice, process severity, and preparation specificity. Extraction technologies were evaluated not only by concentration or yield, but also by selectivity, solvent and energy demand, downstream separation, residue control, scalability, and product use. Processing data were distinguished as content, true retention, extractability, or in vitro bioaccessibility. True retention denotes the proportion of the initial mass of a constituent remaining after processing and is calculated using both constituent concentration and total product mass before and after processing. Clinical findings were summarized with design, sample size, comparator, duration, and principal limitations. Because the search was iterative and no preregistered protocol, exhaustive dual screening, or formal risk-of-bias instrument was used, the review should not be interpreted as a Preferred Reporting Items for Systematic Reviews and Meta-Analyses (PRISMA)-compliant systematic inventory. This limitation is stated to preserve transparency while allowing a broad, mechanism- and technology-focused synthesis.

## 3. Chemical Composition, Phytochemical Profiling, and Quality Assessment

The genus *Cucurbita* includes several economically important species, particularly *C. maxima*, *C. moschata*, *C. pepo*, and *C. ficifolia*, whose fruits are valued for their nutritional quality and chemical diversity. Although the pulp is the principal commercial product, pumpkin processing also generates substantial quantities of peel, rind, seeds, seed coats, defatted seed cake, and pomace. These fractions have complementary compositions: the pulp is particularly rich in carotenoids and polysaccharides, the seeds contain concentrated proteins and lipids, and the peel provides carotenoids, phenolic compounds, and structural polysaccharides. Their principal constituents, representative compositional features, preferred characterization markers, and potential valorization routes are summarized in Table 1. Their composition varies with species, cultivar, maturity, growing conditions, storage, processing, extraction procedure, and analytical method. Direct comparisons therefore require attention to whether concentrations are reported on a fresh- or dry-weight basis and whether they refer to whole tissues or isolated extracts [5,9].

### 3.1. Pulp: Polysaccharides and Carotenoids

The pulp constitutes the largest edible fraction of the pumpkin fruit and provides carbohydrates, dietary fiber, minerals, and several classes of bioactive compounds. Potassium, phosphorus, and magnesium are among its principal mineral constituents. The flesh also contains structurally diverse polysaccharides. In *C. moschata*, optimized ultrasound-assisted aqueous extraction followed by ethanol precipitation yielded 16.04 ± 0.53% crude extract. Yield was determined gravimetrically as the mass of the freeze-dried precipitate divided by the starting pumpkin powder mass, multiplied by 100. This value describes the recovery of a crude polysaccharide fraction and should not be interpreted as total polysaccharide content [25].

Their molecular weight, monosaccharide composition, branching pattern, and degree of esterification influence their physicochemical and biological properties. Additional carbohydrates identified in pumpkin pulp include sucrose, myo-inositol, and D-chiro-inositol, although their concentrations vary considerably among species and cultivars [9,19].

Carotenoids are responsible for the characteristic yellow-to-deep-orange coloration of pumpkin pulp. The carotenoid profile varies among species and cultivars, with α-carotene, β-carotene, lutein, and zeaxanthin among the major pigments reported in pumpkin pulp [20]. In the pulp of 11 *C. maxima* cultivars, Kulczyński and Gramza-Michałowska reported concentrations of 38.67–115.29 μg/g dry weight (DW) for β-carotene, 87.20–388.79 μg/g DW for lutein, and 19.57–192.53 μg/g DW for zeaxanthin [18]. The relative proportions of individual pigments are also important because α- and β-carotene possess provitamin A activity, whereas lutein and zeaxanthin are non-provitamin A xanthophylls [18,20].

Postharvest storage and thermal processing can modify both the concentration and analytical extractability of pulp constituents. During prolonged storage, respiration and cell-wall metabolism can alter sugars, starch, and pectic polysaccharides, although the direction and magnitude of these changes depend on cultivar and storage conditions. Boiling or steaming can disrupt cellular structures and increase carotenoid extractability, whereas intense or prolonged heating may promote carotenoid isomerization and oxidative degradation. Processing may therefore increase measured carotenoid recovery or simulated bioaccessibility without increasing the total amount originally present. Pumpkin tissues also contain peroxidase and other endogenous enzymes relevant to blanching and process control; these proteins should be distinguished from low-molecular-weight phytochemicals [12,26].

### 3.2. Seeds and Seed Oil

Pumpkin seeds and kernels are among the most nutrient-dense fractions of the fruit. Depending on species, cultivar, dehulling, and analytical basis, they contain approximately 14–38% protein, 22–55% lipids, and appreciable amounts of dietary fiber. Their proteins provide a broad spectrum of amino acids, including the essential amino acid tryptophan. The seeds also contain cucurbitine, a non-protein amino acid identified in *Cucurbita* seeds [19,23].

The lipid fraction of the seed is dominated by linoleic and oleic acids, followed by palmitic and stearic acids. Unsaturated fatty acids commonly represent more than 70% of the total fatty acid content, although substantial variation occurs among genotypes and production regions. Pumpkin seed oil also contains tocopherols, whose distribution is cultivar-dependent. γ-Tocopherol frequently predominates in *C. pepo* oils, whereas relatively high δ-tocopherol concentrations have been reported in particular *C. maxima* cultivars. Phytosterols, including β-sitosterol, spinasterol, and stigmastatrienol, together with squalene, constitute additional components of the unsaponifiable fraction, the minor fraction of the oil that remains after alkaline saponification [23,27,28].

Whole seeds and seed-derived flours provide minerals such as zinc, magnesium, iron, and selenium. Pumpkin seeds also contain phenolic compounds, including p-hydroxybenzoic, caffeic, ferulic, vanillic, and protocatechuic acids, together with tyrosol and luteolin. Phytates and oxalates may influence mineral bioaccessibility, but their nutritional significance depends on concentration, processing, and serving size. Defatted seed cake retains substantial protein, fiber, and phenolic compounds and consequently represents a promising substrate for protein concentrates and other value-added food products [21,22].

### 3.3. Peels, Rinds, and Pomace

Pumpkin peels and rinds are frequently discarded despite containing concentrated pigments, phenolic compounds, and dietary fiber. The terms “peel,” “rind,” and “outer pericarp” may denote different tissue depths and should therefore be defined when studies are compared. In several cultivars, the peel contains higher concentrations of β-carotene and total carotenoids than the pulp, with differences of up to approximately tenfold reported in individual studies. Because peel represents a smaller proportion of total fruit mass, concentration per gram should be distinguished from its contribution to the total carotenoid content of the whole fruit [20,22].

The peel is also rich in cellulose, hemicellulose, and pectic polysaccharides. Peel-derived fiber and pectin fractions exhibit water-binding, gel-forming, bile-acid-binding, and fermentability properties that support their potential use as functional food ingredients. Amino acids, including aspartic acid, glutamic acid, and tryptophan, and flavonoids such as kaempferol have also been reported [5]. Saavedra et al. reported radical-scavenging activity of *C. pepo* peel extracts in the 2,2-diphenyl-1-picrylhydrazyl (DPPH) assay. In the same study, in vitro antibacterial testing by disk diffusion using extracts at 10 mg/mL produced no inhibition zones against the four bacterial strains examined [21].

Pomace generated during juice or puree production contains variable proportions of residual pulp, peel, and seeds and therefore combines soluble and insoluble fiber with residual carotenoids and phenolic compounds. Its heterogeneous composition makes characterization and standardization of tissue proportions, moisture, and particle size essential before its application in food, feed, or extraction processes [5].

### 3.4. Flowers, Leaves, and Bioactive Proteins

Pumpkin flowers constitute an edible but underutilized tissue containing polyphenols, flavonoids, carotenoids, and minerals. Their composition varies with species, cultivar, developmental stage, extraction procedure, and flower sex. Leaves and other vegetative tissues have received less systematic investigation than fruits and seeds, making broad comparisons difficult [5].

*Cucurbita* tissues also contain several defense-associated proteins and enzymes. Seeds of *C. moschata* yield α- and β-moschins, arginine/glutamate-rich proteins with translation-inhibiting activity. Separately, basic polypeptides with in vitro antifungal activity have been isolated from *C. maxima* seeds and designated P2249, P4650, and P11696 according to their molecular masses (approximately 2.2, 4.7, and 11.7 kDa, respectively). Defense-related proteins have also been identified in leaves, phloem exudates, developing fruits, and germinating seeds. In addition, proteolytic enzymes isolated from *C. ficifolia* and stable peroxidases obtained from pumpkin pulp have attracted interest for food and biotechnological applications. While these proteins and endogenous enzymes fall outside the strict definition of low-molecular-weight phytochemicals, they represent highly valuable, complementary bioactive constituents [5,6].

### 3.5. Cucurbitacins: Distribution and Safety Considerations

Cucurbitacins are intensely bitter, highly oxygenated tetracyclic triterpenoids characterized by a cucurbitane skeleton. They function primarily as plant defense metabolites and occur in free or glycosylated forms, with their distribution influenced by species, genotype, tissue, and developmental stage. Wild and non-domesticated *Cucurbita* species, including *C. texana* and *C. andreana*, may accumulate substantial concentrations of cucurbitacins B, D, E, and I. By contrast, modern cultivars of *C. maxima*, *C. moschata*, and *C. pepo* have generally been selected for low cucurbitacin concentrations in edible tissues [5,18].

Nevertheless, unusually bitter cultivated fruits should not be consumed because sporadic cucurbitacin accumulation can cause acute gastrointestinal toxicity, including abdominal pain, vomiting, and severe or bloody diarrhea. The low concentrations normally found in non-bitter cultivated pumpkins should therefore not be generalized to intensely bitter fruits [29].

Purified cucurbitacins have demonstrated antiproliferative, pro-apoptotic, and anti-inflammatory activities in experimental models and consequently remain of interest as potential lead compounds for drug development. These findings do not establish the therapeutic efficacy or safety of cucurbitacin-containing foods or unstandardized extracts. Their variable distribution and dose-dependent toxicity require a clear distinction between nutritional exposure and controlled pharmacological investigation [5].

### 3.6. Analytical Profiling and Extract Quality Assessment

Chemical characterization should extend beyond aggregate assays. Carotenoids are most informatively resolved by high-performance liquid chromatography with diode-array detection (HPLC-DAD) or liquid chromatography–mass spectrometry (LC-MS) with isomer- and ester-aware identification; phenolic acids and flavonoids require chromatographic fingerprints and authentic standards where available; fatty acids, sterols, and volatile compounds are commonly profiled by gas chromatography (GC)-based methods; and tocopherols can be quantified by high-performance liquid chromatography (HPLC) with fluorescence or mass-spectrometric detection. For polysaccharides, biological interpretation requires molecular-mass distribution, monosaccharide composition, linkage or methylation analysis, and structural spectroscopy rather than total carbohydrate alone. Protein-rich fractions similarly benefit from amino-acid analysis, electrophoresis or proteomics, digestibility, and allergen assessment [20].

Quality assessment should connect the analytical profile to a reproducible material. Studies should report authenticated species and, where available, cultivar, anatomical fraction, maturity, origin, storage history, pretreatment, extraction yield, concentration basis, and quantified marker compounds. Depending on the matrix and intended application, additional quality attributes include chromatographic fingerprints, batch variability, water activity, oxidation indices, residual solvents, pesticides, mycotoxins, microbial quality, and stability under intended storage conditions [5,20].

Concrete applications of metabolomic profiling have been reported for *Cucurbita* matrices. Miljić et al. applied untargeted UHPLC–Orbitrap mass spectrometry to fruit pulp from 20 accessions comprising 11 *C. maxima* and nine *C. moschata* accessions [30]. Their lipidomic workflow putatively annotated 44 terpenoids and used multivariate analysis to investigate compositional differences among the samples, illustrating its potential for selecting candidate markers of botanical variation. Abdelmonsef et al. combined UPLC–QqQ–MS and GC–MS to characterize *C. pepo* flesh, peel, seeds, juice, and seed oil [31]. They related metabolite profiles to in vitro α-amylase and α-glucosidase inhibition using chemometric analysis, providing an example of how chemical profiling can guide the prioritization of candidate bioactive constituents.

Proton nuclear magnetic resonance (^1^H NMR) profiling has also been used to monitor changes during storage. Di Matteo et al. characterized sugars, amino acids, and organic acids in *C. moschata* samples stored in different commercial packaging films [32]. After 11 days, packaged samples showed increased sucrose and decreased malic acid relative to fresh material, together with differences in amino acid preservation among the packaging systems. These findings illustrate the value of NMR profiling for monitoring compositional changes during storage and comparing the effects of different packaging conditions. A further application concerns product authenticity: Schwarz et al. used untargeted UHPLC–QTOF–MS/MS to select triacylglycerol markers of pumpkin seed oil adulteration and transferred the marker panel to a targeted triple-quadrupole MS/MS workflow [33]. The resulting method detected adulteration at levels as low as 3% (*w*/*w*) under the conditions tested.

Together, these studies illustrate complementary uses of metabolomics in botanical characterization, monitoring of storage effects, bioactivity screening, and authenticity testing. Analytical coverage nevertheless depends on extraction conditions and the measurement platform. Putative metabolite annotations should be distinguished from identities confirmed using authentic standards, while candidate markers and classification models require validation in independent sample sets before routine application. Associations between metabolite profiles and biological assays can guide further experiments, but attribution of activity to individual constituents requires direct functional testing.

### 3.7. Comparative Perspective and Valorization Potential

The anatomical fractions of *Cucurbita* possess complementary compositional profiles. Pulp is characterized principally by carotenoids and polysaccharides; seeds by proteins, unsaturated lipids, tocopherols, phytosterols, and phenolic compounds; and peels and pomace by carotenoids, structural polysaccharides, and residual phenolics. Flowers, leaves, and germinating tissues provide additional phenolic compounds, proteins, and enzymes, although their composition is less comprehensively characterized. Cucurbitacins occupy a distinct position as both specialized defense metabolites and important toxicological markers.

This diversity supports the integrated use of *Cucurbita* tissues as sources of food ingredients, feed materials, and candidate bioactive compounds. Effective valorization nevertheless depends on rigorous characterization and standardization of the starting material. Moreover, biological activity observed in vitro or in animal models should be distinguished from demonstrated clinical efficacy. A composition-centered approach therefore provides the chemical foundation for the subsequent evaluation of processing-induced transformations, mechanisms of action, safety, and translational potential.

## 4. Extraction Technologies and Solvents

Efficient recovery of bioactive constituents from *Cucurbita* biomass is a central operation in the valorization of seeds, peels, fibrous pulp and pomace. The suitability of an extraction process, however, cannot be judged from extraction yield alone. Target-compound polarity, matrix structure, selectivity, solvent and energy consumption, chemical stability, downstream purification, regulatory acceptability and intended product use all influence process selection.

**Figure 1:**
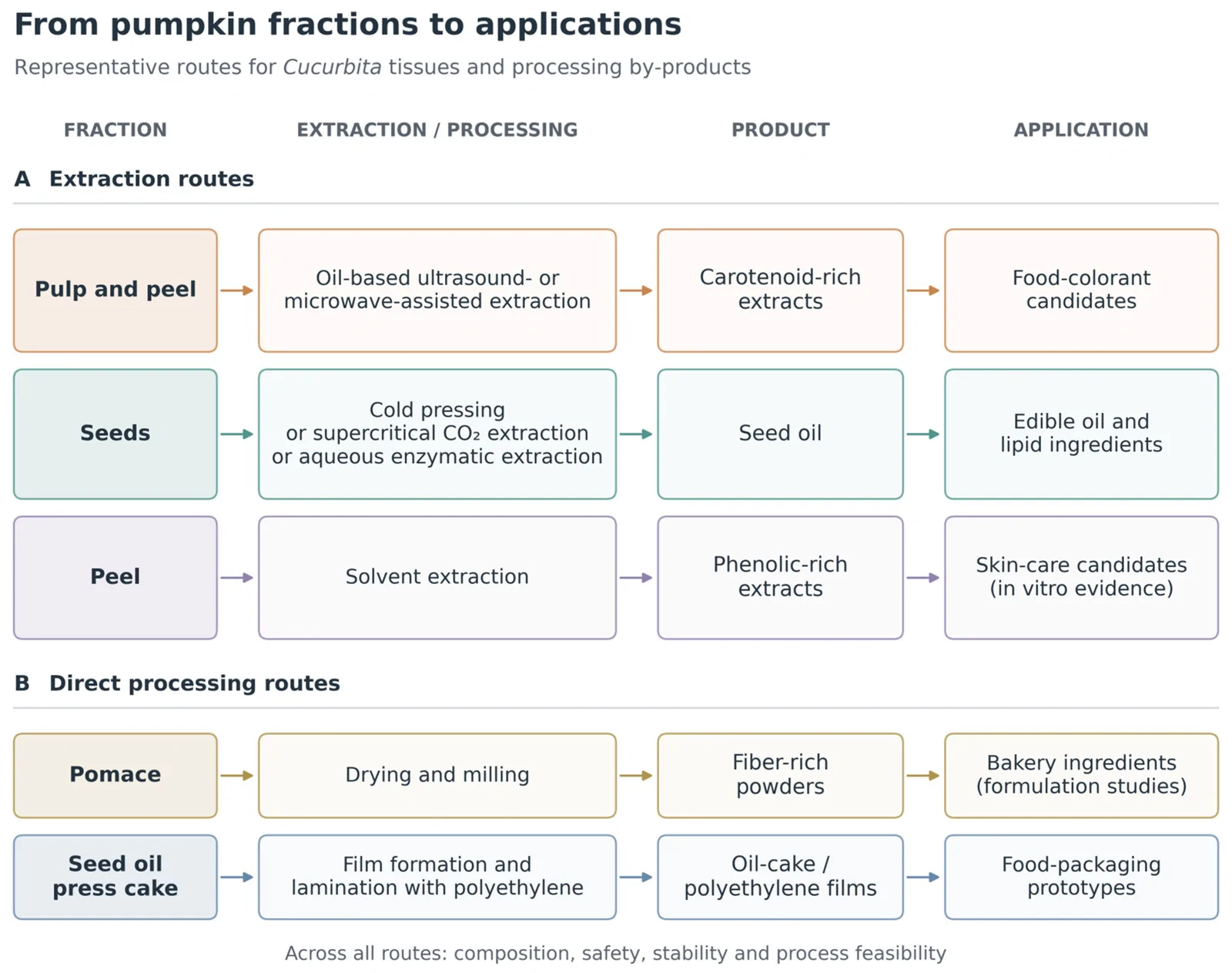
Representative routes linking *Cucurbita* tissues and processing by-products to extraction or processing methods, resulting products, and applications. (**A**) Selected extraction routes for carotenoid-rich extracts, seed oil, and phenolic-rich peel extracts [13,34,35,36]. (**B**) Direct processing of pomace into fiber-rich powders and seed oil press cake into composite packaging films [37,38].

Conventional solvent extraction and mechanical pressing remain important reference and production methods, whereas supercritical fluids, DES, enzyme-assisted aqueous processing and physical intensification technologies offer alternative routes with different benefits and constraints. The following subsections compare these approaches, with emphasis on evidence generated in *Cucurbita* matrices and on the practical trade-offs that determine scale-up potential. Figure 1 provides a schematic overview of representative routes linking pumpkin tissues and processing by-products to extraction or processing methods, resulting products, and applications. The extraction technologies are discussed in the following subsections, while product applications are addressed later in the review.

### 4.1. Conventional Extraction and Mechanical Pressing

Soxhlet extraction, maceration, heat-reflux extraction and stirred solid–liquid extraction are widely used at laboratory scale to characterize lipids, pigments and phenolic compounds in pumpkin seeds and processing by-products. Aqueous ethanol and other polar protic solvents are generally appropriate for phenolic acids and flavonoids, whereas methanol is mainly used for analytical extraction [39]. Non-polar solvents such as hexane and petroleum ether are effective for fixed (nonvolatile) oils and other lipophilic constituents, while acetone and mixed-solvent systems are frequently used for carotenoid analysis. In commercial food processing, solvent selection is more restrictive because food-grade status, permitted residues, solvent recovery and worker safety must also be considered [26].

Mechanical pressing provides a solvent-free route to virgin pumpkin seed oil and requires comparatively little downstream purification. Its recovery efficiency nevertheless depends strongly on seed moisture, thermal preconditioning or roasting, particle size, screw configuration, and applied pressure. In a recent expeller study of *C. moschata* genotypes, press-cake residual oil ranged from approximately 14% to 18% on a dry-mass basis, illustrating that a meaningful fraction of the lipid can remain after pressing [39]. This residual fraction may be recovered in a secondary extraction step or valorized together with the protein- and fiber-rich cake.

The principal disadvantages of conventional solvent extraction are long processing times, solvent consumption, energy demand for heating and solvent recovery, and the generation of solvent-containing waste streams. Prolonged exposure to heat, oxygen and light may promote oxidation or isomerization of carotenoids and degradation of polyunsaturated lipids, although the extent of damage depends on the actual temperature–time history and oxygen control. Despite these limitations, conventional methods remain robust and economically important; they should therefore be treated as benchmarks rather than as uniformly obsolete technologies [26].

### 4.2. Supercritical CO_2_ Extraction

Above its critical point (31.1 °C and 7.38 MPa), carbon dioxide behaves as a supercritical fluid with gas-like diffusivity and liquid-like solvating capacity. Pumpkin seed oil extraction is commonly investigated at approximately 40–60 °C and 20–40 MPa. Changes in pressure and temperature alter CO_2_ density and therefore extraction selectivity. Because SC–CO_2_ is predominantly non-polar, it is well suited to triacylglycerols, phytosterols, tocopherols and carotenoids; polar phenolics generally require a modifier such as ethanol or a separate extraction stage [40].

Evidence from pumpkin seed oil illustrates a trade-off between oil yield and phytosterol concentration in the recovered oil. Hrabovski et al. reported that extraction at 40 °C and 40 MPa produced an oil yield of approximately 36%, compared with approximately 43% for hexane and 45% for petroleum ether. The SC–CO_2_ oil had a total phytosterol concentration, defined as free plus esterified sterols, of 294 mg/100 g oil, approximately 30% higher than that of the hexane extract and 20% higher than that of the petroleum-ether extract [34]. These differences describe phytosterol enrichment per unit mass of oil and do not, by themselves, demonstrate greater phytosterol recovery from seeds. The amount recovered per unit mass of seeds depends on both oil yield and phytosterol concentration; expressing recovery as a percentage additionally requires the initial phytosterol content of the seeds. A later comparison reported differences in individual phytosterol concentrations among recovered oil fractions, while fatty-acid profiles were broadly similar between the main SFE–CO_2_ oil fraction, hexane-extracted oils, and cold-pressed oils [41]. That study also observed orange-yellow SFE oil and a brownish-green hexane extract, consistent with differences in co-extracted constituents; color alone does not establish thermal degradation.

SC–CO_2_ reduces exposure to organic solvents and allows extract recovery by depressurization, which is attractive for high-value food, nutraceutical and cosmetic ingredients. Its limitations include the capital cost of high-pressure equipment, energy required for CO_2_ compression and recirculation, batch throughput, and limited recovery of polar compounds without a co-solvent [40]. Consequently, SFE is most compelling when improved selectivity, solvent-free product specifications or higher concentrations of unsaponifiable constituents in the recovered oil justify the additional process cost; it is not necessarily the highest-yield route for bulk oil production.

### 4.3. Deep Eutectic Solvents

DES are formed by combining a hydrogen-bond acceptor with a hydrogen-bond donor in a ratio that produces a substantial melting-point depression. Systems prepared from naturally occurring constituents are often termed natural deep eutectic solvents (NADES). Their polarity, viscosity and water affinity can be adjusted through component selection and molar ratio, enabling hydrophilic formulations for phenolics and hydrophobic formulations for carotenoids and other lipophilic compounds. Their very low vapor pressure can reduce volatile-organic emissions, but this property does not by itself establish low toxicity or low overall environmental impact [42].

Pumpkin by-products provide several proof-of-concept examples. Stupar et al. combined ultrasound with a switchable hydrophobic NADES based on caprylic and capric acids at a 3:1 molar ratio to recover β-carotene from pumpkin material [43]. More recently, screening of nine natural hydrophobic DES formulations identified DL-menthol/lactic acid (1:2) as the most effective tested combination for pumpkin-peel carotenoids. Under matched screening conditions, the formulation recovered approximately 94% of the β-carotene obtained with acetone; however, this corresponded to only about 25% of the carotenoid content estimated by exhaustive acetone extraction [44]. Reporting both comparators prevents the matched-solvent result from being interpreted as near-quantitative recovery.

DES-based extraction is therefore promising but remains less mature than its low volatility and tunability may suggest. High viscosity can restrict mass transfer and increase pumping requirements, while water addition may change solvent structure and selectivity. Separation of the target compound from the DES, solvent regeneration, residual-solvent specifications, sensory effects, toxicological characterization and food-regulatory acceptance must be evaluated for each formulation. These downstream issues are particularly important when the DES is not intended to remain as part of the final ingredient.

### 4.4. Enzyme-Assisted Aqueous Extraction

EAAE uses water together with cell-wall- or storage-matrix-degrading enzymes, including cellulases, pectinases, hemicellulases and proteases. Enzyme selection should reflect the matrix: pectinases and cellulases facilitate disruption of peel and pulp tissues, whereas proteases can help release intracellular lipid-storage droplets, known as oil bodies, from seed material. Hydrolysis weakens the structural matrix and can improve the release of oil, carotenoids and proteins under relatively mild temperature and pH conditions [45].

For pumpkin seeds, aqueous enzymatic processing offers the additional possibility of recovering oil and protein-rich fractions within the same biorefinery. Konopka et al. reported that pumpkin oil obtained by aqueous enzymatic extraction was richer in sterols, squalene and tocopherols than cold-pressed oil, but it showed lower oxidative stability [35]. This finding illustrates that higher concentrations of selected micronutrients in the recovered oil do not automatically translate into greater oxidative stability, which is also influenced by pro-oxidants, endogenous enzymes, interfacial area and the efficiency of water removal.

The major technical difficulty is formation of stable oil–water–protein emulsions, which can reduce free-oil recovery and require centrifugation, membrane separation, demulsification or subsequent mechanical expression. Enzyme cost, hydrolysis time, pH adjustment, microbial control and wastewater generation also affect process feasibility [45]. EAAE is therefore particularly attractive when several product streams—such as oil, protein hydrolysates and fiber—are recovered, rather than when oil yield is the only process objective.

### 4.5. Ultrasound- and Microwave-Assisted Extraction

Ultrasound-assisted extraction (UAE) improves mass transfer through acoustic cavitation. Bubble collapse near plant particles generates shear, micro-jets and local turbulence that can disrupt tissue, reduce boundary-layer resistance and improve solvent penetration. UAE can shorten extraction time and can be combined with aqueous ethanol, edible oils or DES. Its performance depends on acoustic power density, frequency, probe or bath geometry, solid loading and temperature control; results obtained in small probes cannot be transferred to large vessels on treatment time alone [46].

Microwave-assisted extraction (MAE) uses dipolar rotation and ionic conduction to heat microwave-absorbing components within the solvent and plant matrix. Rapid internal heating can increase pressure within hydrated cells, accelerate matrix disruption and lower solvent viscosity. The technique is particularly effective with water–ethanol mixtures and other sufficiently polar media, whereas non-polar solvents couple poorly with microwave energy unless an absorbing phase is present. Excessive power or inadequate mixing can create hot spots and degrade heat-sensitive compounds [47].

In pumpkin peel and pulp, Sharma and Bhat reported nearly twofold higher total carotenoid concentrations for UAE and MAE performed in corn oil than for a conventional hexane/isopropanol procedure [13]. Because both the physical treatment and extraction medium differed, the gain cannot be attributed solely to ultrasound or microwave energy. The study nevertheless demonstrates the potential of directly producing a carotenoid-enriched edible oil. At larger scale, UAE requires control of cavitation uniformity, oxidation and probe erosion, whereas MAE requires attention to penetration depth, temperature distribution, pressure and continuous material handling [46,47].

### 4.6. Hybrid and Emerging Processes

Hybrid strategies combine complementary mechanisms rather than assuming that a single treatment will overcome all mass-transfer barriers. Ultrasound-assisted enzymatic extraction and microwave-assisted enzymatic extraction may reduce the severity required from either treatment alone: enzymatic hydrolysis weakens the cell-wall matrix, after which acoustic or thermal disruption can improve solvent access. The magnitude of synergy nevertheless depends on enzyme compatibility, temperature, shear exposure and process sequence, and it should be demonstrated against properly matched single-treatment controls [48].

HPH and PEF are also being explored as pretreatments for *Cucurbita* products. In *C. maxima* cv. Hokkaido puree, HPH and enzyme-assisted processing increased carotenoid transfer to the juice fraction to approximately 90–98% and 72–90%, respectively, whereas PEF produced no significant improvement [26]. These results show both the potential of mechanical cell disruption and the matrix dependence of emerging non-thermal technologies. They also concern whole-pumpkin puree rather than pomace extraction, so their direct transfer to industrial by-products requires validation.

Integration with downstream separation is essential when evaluating hybrid processes. Increased cell disruption may improve apparent extraction while simultaneously releasing pectin, protein or fine particles that complicate clarification, filtration or emulsion breaking [48]. Process comparisons should therefore include not only extract concentration, but also mass yield, purity, energy consumption, solvent recovery, separability and stability of the final ingredient. The suitable targets, principal advantages, limitations, and development status of the extraction technologies discussed above are summarized in Table 2.

### 4.7. Process Selection and Outlook

Process selection should match the target fraction and downstream product requirements. SC–CO_2_ is suited to high-value lipophilic extracts when selectivity and solvent-free specifications justify high-pressure processing. EAAE offers an option for recovering oil alongside protein and fiber streams. UAE and MAE intensify extraction, with performance governed by solvent, reactor geometry and temperature control; HPH and PEF can serve as pretreatments. For DES and NADES, downstream separation, toxicological evaluation and regulatory acceptability remain key considerations.

Environmental and economic evidence is available for selected *Cucurbita* valorization routes. Massironi et al. compared water and ethanol/water extraction routes for cucurbitine from *C. pepo* seed hulls. Using laboratory process inventories and a functional unit of extracts containing 1 kg of cucurbitine, their LCA estimated a 37% lower carbon footprint for the water-extraction route [49]. Konopka et al. provided a simplified cost estimate for aqueous enzymatic oil extraction [35]. Noman et al. conducted a TEA of microbial enzyme production using pumpkin peels as a fermentation substrate [50]. This latter assessment concerns microbial bioconversion; comparative LCA and TEA of native-compound extraction at industrial scale remain limited within the evidence reviewed here.

**Figure 2:**
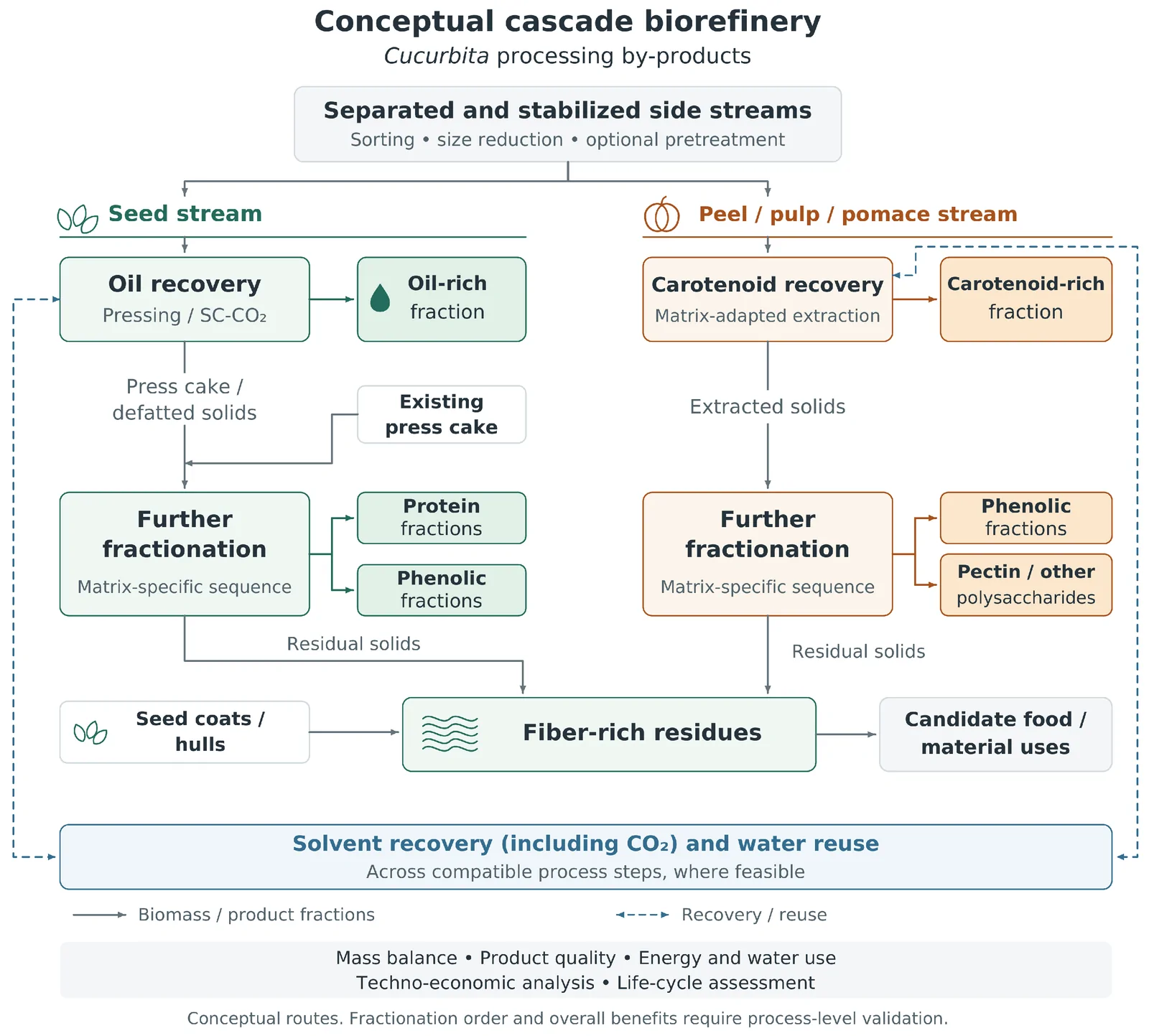
Conceptual cascade biorefinery for the valorization of *Cucurbita* processing by-products. Separated and stabilized seed and peel/pulp/pomace streams undergo matrix-specific recovery of oil-rich or carotenoid-rich fractions, followed by further fractionation of the remaining biomass. Existing press cake can enter downstream fractionation directly, while seed coats and hulls can enter the fiber-rich stream. Solid arrows represent biomass and product flows; dashed bidirectional links indicate recovery and reuse of process solvents, including CO_2_ where applicable, and water where feasible. The fractionation sequence is illustrative and should be optimized for the matrix, target products and downstream compatibility. Candidate food and material uses require suitable product quality and safety. Economic and environmental benefits require process-level validation. SC-CO_2_, supercritical carbon dioxide.

Table 3 summarizes representative extraction conditions and quantitative outcomes, retaining each study’s definition of yield, recovery, or extract concentration. Future comparisons should use characterized matrices and report target-compound recovery alongside resource inputs. Figure 2 outlines a conceptual, matrix-specific cascade biorefinery for *Cucurbita* processing by-products. Its evaluation should consider the combined product outputs, downstream separation requirements, solvent recovery, and resource demands of the full process.

## 5. Effects of Processing on Composition, Bioaccessibility, and Quality

Processing affects pumpkin through several mechanisms that should not be treated as equivalent. A lower measured concentration can represent true chemical degradation, leaching into a discarded liquid phase, or dilution, whereas an apparent increase can arise from water removal, cellular disruption, improved analytical extraction, or formation of assay-reactive products. Digestive bioaccessibility is a separate outcome again: a compound may become more accessible from the food matrix even when its total content decreases. Consequently, processing effects should be interpreted using a common wet- or dry-matter basis, and preferably through true-retention or mass-balance calculations [12].

The direction and magnitude of change also depend on *Cucurbita* species and cultivar, maturity, anatomical fraction, particle size, oxygen exposure, processing medium and the complete time–temperature–pressure history. Results obtained for flesh, peel, juice, puree, seeds, oil and dried powder are therefore not directly interchangeable. The following discussion focuses on representative food-processing operations and separates compositional retention from extractability, bioaccessibility, sensory quality and microbial stability.

### 5.1. Mechanical Preprocessing, Blanching, and Freezing

Peeling, cutting, comminution, pureeing and juicing alter composition before heat or fermentation is applied. Peeling removes outer tissues that may contain a different concentration of carotenoids, phenolics and fiber than the flesh. Cutting and pureeing reduce particle size and release intracellular constituents, which can improve dispersibility and analytical extraction but also increase contact with oxygen and endogenous enzymes. Juicing partitions insoluble fiber and particle-associated compounds between the liquid and pomace; a lower concentration in clarified juice may therefore reflect physical separation rather than molecular destruction [22].

Blanching is commonly used before freezing or drying to suppress polyphenol oxidase, peroxidase and other endogenous enzymes. Its benefit must be balanced against thermal degradation and the leaching of water-soluble compounds. In a comparison of the pumpkin cultivars Butternut Rugosa (*C. moschata*), Nelson F1 (*C. maxima*), and Hokkaido ‘Uchiki Kuri’ (*C. maxima*), blanching before freezing reduced subsequent losses relative to freezing without blanching, although the magnitude of protection was cultivar-dependent [51]. This finding supports blanching as a useful pretreatment under the tested conditions rather than as a universal requirement with a single optimal schedule.

Freezing slows microbial and chemical deterioration but does not eliminate reactions during storage. Ice-crystal formation can disrupt cell walls, producing softening and drip loss after thawing, while residual enzyme activity and oxygen in the package can continue to affect color and labile compounds [52]. Freezing rate, storage temperature, temperature fluctuations, package permeability and thawing method therefore need to be reported when frozen pumpkin is compared with fresh material.

### 5.2. Fermentation and Microbial Biotransformation

Controlled fermentation can preserve pumpkin-derived products while modifying acidity, flavor, rheology and the analytical profile of bioactive constituents. Lactic acid bacteria (LAB) and yeasts consume soluble sugars and generate organic acids and volatile metabolites. Strain-specific glycosidases and other enzymes may release phenolic aglycones or transform individual phenolic acids; at the same time, microorganisms may consume phenolics, carotenoids or organic acids. Total phenolics and chemical antioxidant assays can therefore increase or decrease according to the organism, substrate, pretreatment, fermentation time and oxygen exposure [53].

Pumpkin juice provides a suitable example of this variability. Janiszewska-Turak et al. compared five LAB strains in heat-treated and untreated juice [53]. Acidification occurred with all strains, and *Latilactobacillus sakei* showed comparatively good adaptation, but the authors also concluded that cell growth required further optimization. The study did not support a single strain as superior for every chemical and quality outcome. Viable LAB counts should therefore be reported as such; the term probiotic is appropriate only when the specific strain and dose have demonstrated a health benefit in the intended host.

Mixed-culture fermentation can generate a broader metabolic and sensory response. In a laboratory study of pumpkin juice that had first been steamed and sterilized, a consortium of *Saccharomyces cerevisiae*, *Lactobacillus helveticus* and *Lactobacillus delbrueckii* subsp. *bulgaricus* was incubated at 35 °C for 27 h. The treatment reduced pH from 6.01 to 3.81, produced a viable count of approximately 7.37 log CFU/mL, increased measured total phenolics and changed the volatile profile toward a higher proportion of esters and alcohols, while the authors reported only a small instrumental color change [54]. These outcomes describe one defined consortium and pretreatment sequence and should not be generalized to spontaneous fermentation or other pumpkin matrices.

De novo formation of a metabolite is particularly organism-dependent. Fermentation of pumpkin juice with the photosynthetic bacterium *Rhodobacter sphaeroides*, for example, has been investigated as a route to coenzyme Q10 enrichment [55]. This observation cannot be attributed to LAB fermentation in general. Similarly, increases in DPPH, Ferric Reducing Antioxidant Power (FRAP) or related assays demonstrate altered in vitro reducing or radical-scavenging capacity, not improved human bioavailability or clinical efficacy.

### 5.3. Thermal Processing, Cooking, and Seed Roasting

Thermal processing produces a balance between tissue disintegration and chemical deterioration. Heating softens pectin-rich cell walls, changes starch and protein structures, and can release carotenoids from chromoplast-associated structures [51]. These changes may improve extractability and incorporation into mixed micelles, small lipid–bile aggregates that disperse carotenoids in intestinal fluid and make them potentially available for absorption. Conversely, long exposure to heat, oxygen and light promotes carotenoid oxidation and isomerization, ascorbic-acid loss and changes in phenolic compounds [12]. The net result depends on both process severity and the physical matrix.

This distinction is illustrated by in vitro digestion data. Koh and Loh found that cooking reduced β-carotene concentration in the tested pumpkin, while deep-frying significantly increased percentage bioaccessibility; the increases after boiling and steaming were not statistically significant [56]. Percentage bioaccessibility was calculated as B (%) = 100 × (a/b), where a is the β-carotene recovered in the micellar fraction after simulated digestion and b is the total β-carotene in the corresponding undigested food sample, both expressed in mg per 100 g of the respective raw or cooked food. For cooked samples, the denominator therefore refers to β-carotene present after cooking and before digestion. The absolute bioaccessible mass for a defined portion is calculated by multiplying its β-carotene content (mg per portion) by B/100. Thus, a higher percentage alone does not necessarily indicate a greater bioaccessible mass. The increase after deep-frying may reflect enhanced micellarization in the presence of added lipid. This finding does not establish deep-frying as the nutritionally optimal method, because oil uptake and the stability of the frying medium must also be considered. It illustrates why concentration, retention, percentage bioaccessibility and absolute bioaccessible mass should be reported separately.

Water-based cooking introduces an additional loss pathway through leaching. In the three-cultivar experiment of Piepiórka-Stepuk et al., immersion cooking was the least favorable treatment for the selected water-soluble bioactive compounds, with losses reaching approximately 80% in the most affected cultivar–compound combinations, whereas steaming was generally more favorable [51]. The conclusion should remain limited to those cultivars and treatment conditions. If the cooking liquid is consumed, as in soup production, leached compounds remain within the meal and should not automatically be counted as dietary losses.

Carotenoid stability is likewise compound- and cultivar-specific. During the production of purees from *C. moschata* ‘Menina Brasileira’ and *C. maxima* ‘Exposição’, Provesi et al. reported true retentions above 75% for the major provitamin A carotenes after processing, while xanthophylls such as lutein and violaxanthin were less stable [12]. Only low concentrations of cis-β-carotene isomers were detected. These findings do not support treating a single kinetic constant or percentage loss as a general property of pumpkin. Instrumentally measured color changes are also indirect and may reflect not only changes in carotenoid content but also browning, moisture, pH, particle size, and light scattering. Therefore, color measurements should not be interpreted as direct evidence of β-carotene degradation.

Reported increases in total phenolics or antioxidant activity after cooking can result from cell-wall disruption, improved solvent access, concentration changes or the formation of Maillard-reaction products. Dini et al., for example, observed higher measured phenolic content and antioxidant properties after domestic and industrial processing of pumpkin pulp [57]. Such results are analytically meaningful but do not by themselves demonstrate an increase in the mass of native phenolics. Studies should report the calculation basis and, where possible, quantify individual compounds rather than relying only on global colorimetric assays.

Roasting deserves separate consideration because pumpkin seeds contain substantial oil and protein. In *C. pepo* seeds roasted at 120, 160 or 200 °C for 10 min, measured total phenolics, flavonoids and antioxidant activity increased with temperature, while Maillard products and lipid-peroxidation products were also detected; 160 °C gave the most favorable protein-quality outcome within that experimental design [58]. The result illustrates a flavor–functionality–oxidation trade-off and should not be converted into a universal roasting recommendation. Seed moisture, atmosphere, layer thickness and subsequent oil storage can change the optimum substantially.

Commercial pasteurization or sterilization must ultimately be judged against a defined safety target. Autoclaving *C. moschata* puree at 121 °C for 10–30 min reduced the measured microbial load and altered pH, soluble solids, carotenoids and the volatile profile [59]. A reduction in native counts, however, is not equivalent to validated commercial sterility. The target organism or spore, required log reduction, package geometry and storage conditions must be specified for any safety claim.

### 5.4. Pressure-Assisted and Other Intensified Preservation Treatments

High-pressure-assisted thermal processing (HPTP) combines hydrostatic pressure with moderate or elevated initial temperature to increase microbial and enzyme inactivation while limiting treatment time. In Butternut pumpkin puree, García-Parra et al. compared nine combinations of 300, 600 or 900 MPa with initial temperatures of 60, 70 or 80 °C for 1 min [60]. Microbial inactivation increased with pressure and temperature, and most treatments reduced polyphenol oxidase activity while maintaining or increasing extractable carotenoids and phenolics.

Within that experimental design, 600 MPa/70 °C produced the greatest reduction in polyphenol oxidase together with favorable measured bioactive-compound and antioxidant responses. The authors nevertheless indicated that the processed puree required low-temperature storage. The condition should therefore be described as the best-performing treatment in that study, not as a general industrial optimum or proof of commercial sterility. PEF, ultrasound or other pretreatments should likewise be evaluated as separate unit operations unless their combined safety, quality and energy performance has been tested in the same process sequence.

### 5.5. Drying and Dehydration

Drying reduces water activity—the availability of water for microbial growth and chemical reactions—and transport mass, enabling the production of shelf-stable pumpkin slices, flours and by-product powders. It simultaneously concentrates non-volatile solids and exposes the matrix to combinations of heat, oxygen, light and prolonged residence time. Comparisons with fresh material must therefore use a dry-matter basis or true-retention calculation. Otherwise, an apparent increase in protein, fiber, sugars, carotenoids or phenolics may simply reflect water removal [61].

Conventional hot-air drying is accessible and scalable, but shrinkage and long exposure to oxygen can impair color, rehydration, ascorbic acid and carotenoid retention. Freeze-drying generally limits thermal damage and produces an open porous structure, but it does not perfectly preserve fresh tissue: losses may occur during pretreatment, freezing, sublimation and subsequent oxygen-exposed storage, and capital and energy demands are high [61]. In the flesh of seed-type *C. maxima* pumpkins, freeze-dried material showed better color and higher retention of β-carotene and ascorbic acid than hot-air-dried material, whereas far-infrared drying produced the highest measured polyphenol content and radical-scavenging activity [62]. Thus, the preferred drying method depends on the quality attribute being prioritized.

Multi-flash drying applies successive heating–vacuum-pulse cycles that cause rapid flash evaporation and generate a porous product structure. Together with microwave-vacuum drying, this approach can substantially shorten drying time. Monteiro et al. dried pumpkin slices to approximately 0.022 g water/g dry solids and a water activity of 0.438 in 1.28 h by microwave-vacuum drying and 1.32 h by microwave multi-flash drying, compared with 16.33 h for air drying and 16.67 h for freeze-drying [63]. These are substantial time reductions under the reported laboratory conditions. Rehydration was nevertheless temperature-dependent: microwave-vacuum and microwave multi-flash samples performed favourably at 25 °C, whereas freeze-dried samples had the highest rehydration ratio at 80 °C. These findings indicate that the rehydration performance of dried pumpkin slices depends on both the drying method and the rehydration temperature.

Drying-kinetic parameters and model fits depend on cultivar, sample thickness and geometry, pretreatment, air conditions, moisture range, apparatus, and model assumptions [61]. Values obtained under different conditions should therefore not be combined into a single range without harmonized methods, and model selection should be supported by residual analysis and independent validation rather than goodness of fit alone. The principal compositional and structural effects, potential advantages, and interpretive limitations of the processing operations discussed in this section are summarized in Table 4.

### 5.6. Integrated Interpretation and Process Selection

Processing should be evaluated against the intended product outcome: nutrient retention, digestive bioaccessibility, microbial safety, sensory quality, storage stability or ingredient functionality. Tissue disruption can increase carotenoid release, whereas prolonged heat and oxygen exposure can reduce retention. Fermentation modifies acidity, flavor and metabolite composition, while drying combines stabilization with changes in labile nutrients and powder properties.

In addition to the material characterization outlined in Section 3.6, comparisons should specify particle geometry, processing medium, oxygen exposure and complete treatment conditions. Explicit wet- and dry-matter bases, together with mass-balance or true-retention calculations, help separate concentration effects from compound loss. Individual carotenoid and phenolic measurements should complement aggregate assays. Preservation studies should link quality measurements to validated microbial or enzymatic targets and storage performance.

Suitable options differ by product: steaming may limit leaching from pumpkin pieces, controlled fermentation can produce acidified beverages, roasting can be optimized for seed flavor and oil quality, and freeze- or microwave-vacuum drying may suit powders where structure and color are priorities. Energy efficiency should be considered alongside these quality objectives, particularly when selecting drying routes for large volumes of processing residues.

## 6. Biological Activities, Human Evidence, and Translational Priorities

Pumpkin flesh, peel, seeds, seed oil and processing by-products contain chemically diverse constituents, including carotenoids, phenolic compounds, tocopherols, phytosterols, unsaturated fatty acids, proteins, peptides, polysaccharides and minerals. These constituents provide plausible mechanisms for biological activity, but their presence alone does not establish therapeutic efficacy. The evidence base spans chemical antioxidant assays, molecular docking, isolated-enzyme experiments, cultured cells, animal models and a comparatively small set of human studies. Conclusions should therefore be matched to the highest level of evidence actually available for each outcome.

**Figure 3:**
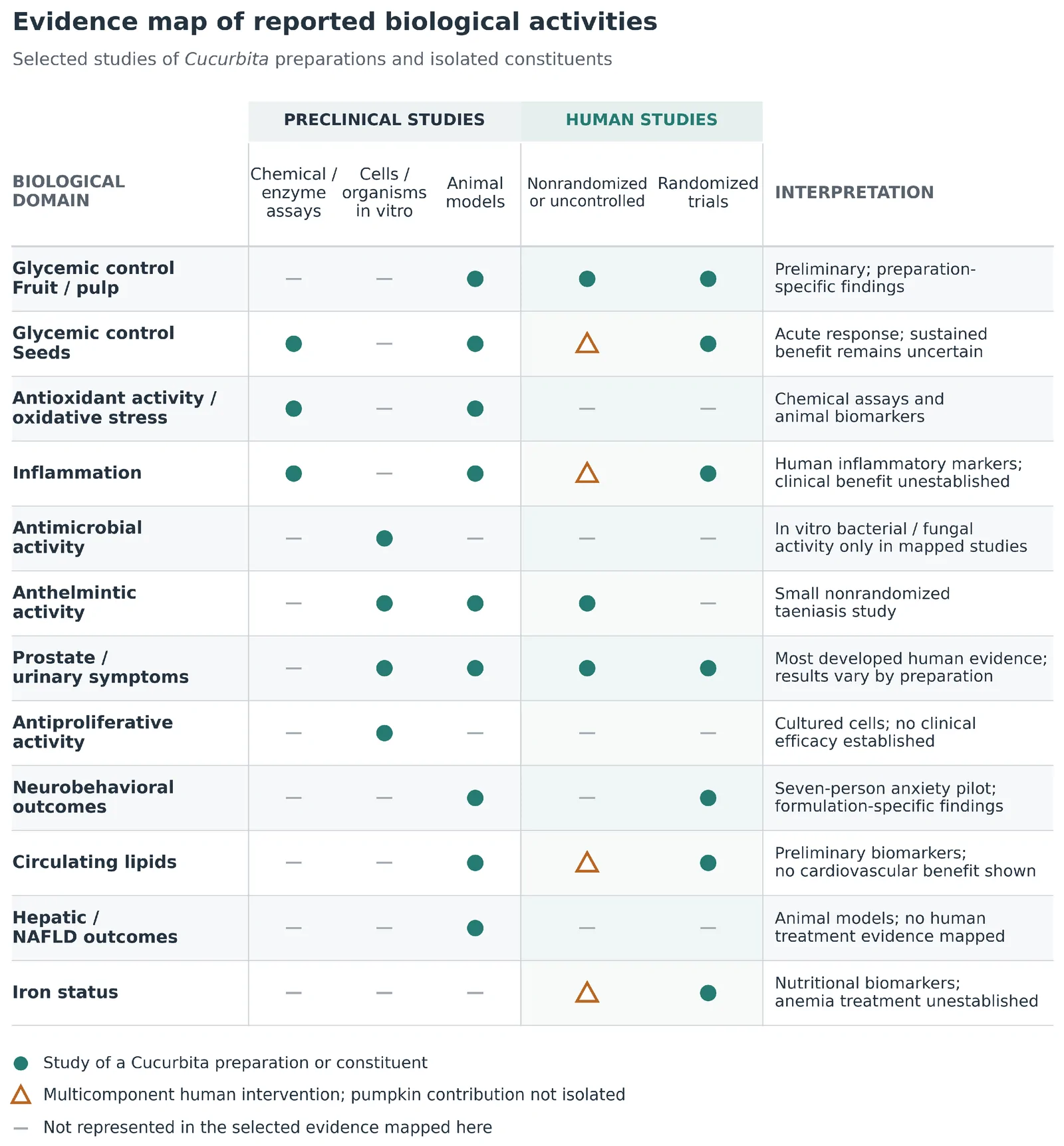
Evidence-level matrix of reported biological activities of *Cucurbita* preparations and isolated constituents. The matrix summarizes representative evidence from chemical and enzyme assays, cells or organisms studied in vitro, animal models, and human studies, distinguishing randomized trials from nonrandomized or uncontrolled designs. It covers metabolic, antioxidant, inflammatory, antimicrobial, anthelmintic, urinary, antiproliferative, neurobehavioral, hepatic, and iron-related outcomes [10,11,64,65,66,67,68,69,70,71,72,73,74,75,76,77,78,79,80,81,82,83,84,85,86,87,88,89]. Filled circles indicate at least one relevant study of a *Cucurbita* preparation or constituent; open triangles indicate evidence limited to multicomponent human interventions in which the pumpkin contribution was not isolated; dashes indicate that no study was mapped in that category within the selected evidence. Symbols indicate study presence, not study counts, demonstrated efficacy, or a formal certainty assessment. The interpretation column highlights preparation-specific findings and the principal limitations of translation to human benefit. In vitro organisms include microbes and helminths. NAFLD, nonalcoholic fatty liver disease.

This distinction is particularly important for *Cucurbita* because species, cultivar, anatomical fraction and preparation are not interchangeable. A purified cucurbitacin from a wild species, a hydroalcoholic seed extract, whole roasted seed, seed oil and a formulated multi-ingredient product can differ greatly in composition, exposure and biological effect. Likewise, a docking score identifies a possible molecular interaction rather than demonstrating that the compound reaches and interacts with the proposed target in vivo, and a change in an oxidative-stress marker in a rodent model does not by itself demonstrate prevention or treatment of human disease. The following synthesis therefore presents proposed mechanisms together with the limitations that govern their interpretation. Figure 3 provides an overview of the study designs supporting the biological activities discussed in the following subsections, distinguishing experimental findings from human evidence and highlighting the principal limitations of their interpretation.

### 6.1. Framework for Interpreting Biological Evidence

Mechanistic studies are useful for generating and refining hypotheses, but they do not by themselves establish clinical efficacy. Enzyme-inhibition assays can demonstrate inhibition of α-amylase, α-glucosidase, 5α-reductase or another target under defined laboratory conditions, but whether the inhibitory concentration is achievable at the relevant site after ordinary dietary intake requires exposure and bioavailability data [90,91]. Molecular docking predicts plausible ligand–target interactions computationally and should not be treated as evidence of biochemical inhibition without experimental validation [92]. Cultured-cell studies add information about uptake, cytotoxicity and signaling, although they cannot reproduce digestion, metabolism, tissue distribution or whole-organism toxicity [90,91].

Animal studies provide evidence that an intervention can affect a biological system, yet many *Cucurbita* experiments use chemically induced disease, high doses and short treatment periods [93]. Their results should be reported as model-specific effects rather than as proof of human treatment. Human efficacy is best assessed in randomized trials with an appropriate comparator, prespecified outcomes, adequate power and a preparation characterized sufficiently for replication. Uncontrolled studies, pre–post comparisons and trials of mixed products can provide preliminary signals but cannot reliably isolate a pumpkin-specific effect [90,91,93].

Claims of synergy require a design that compares the combination with each component and tests an interaction; improvement after a combined intervention is insufficient. Similarly, safety cannot be inferred from the absence of reported adverse events in a small or short study. Throughout this section, terms such as “reduced,” “inhibited” and “improved” refer only to the stated assay, model or clinical endpoint.

### 6.2. Glycemic Control and Metabolic Outcomes

Evidence for glycemic effects has been reported for both fruit/pulp and seed preparations. In streptozotocin-induced diabetic rats, aqueous extracts prepared from dried *C. moschata* and *C. maxima* pulp and administered orally at 500 mg/kg/day for six weeks reduced blood glucose and were associated with changes in serum insulin and pancreatic-islet morphometry, with larger effects reported for the *C. maxima* extract [64]. Because the study used small groups, a single dose, concentrated extracts, and a chemically induced model, its findings do not establish that dietary pumpkin pulp treats diabetes or regenerates pancreatic β-cells in humans.

Limited direct human evidence for fruit and pulp preparations is also available. In a controlled within-participant study, ten adults with type 2 diabetes received a single dose of raw *C. ficifolia* fruit extract (4 mL/kg) or water on separate occasions. Blood glucose decreased from approximately 217 mg/dL at baseline to 151 mg/dL five hours after administration of the fruit extract, whereas no significant change followed water administration [65]. In an eight-week randomized trial, 80 adults with type 2 diabetes were allocated to 100 g/day of green *C. ficifolia* fruit, probiotic yogurt, their combination, or dietary advice alone. Fasting glucose and glycated hemoglobin (HbA1c) decreased in the *C. ficifolia* group relative to the control group [66]. However, the study included only 20 participants per group, was not blinded, had a short follow-up period, and did not provide detailed compositional standardization of the fruit preparation.

A separate uncontrolled before–after study included 20 critically ill diabetic patients who received lyophilized *C. maxima* pulp at 5 g twice daily for three days in addition to insulin. Mean blood glucose decreased from approximately 215 mg/dL during the three days preceding supplementation to 178 mg/dL during supplementation, whereas the reduction in insulin requirement was not statistically significant [67]. The absence of a concurrent control group, the short intervention, critical illness, concomitant treatments, and possible temporal changes in disease severity prevent causal attribution to the pumpkin preparation. Furthermore, findings obtained with *C. ficifolia* or *C. maxima* cannot automatically be generalized to other *Cucurbita* species or pulp preparations.

Seed-derived preparations have also been examined for effects relevant to carbohydrate digestion. An aqueous *C. maxima* seed extract inhibited α-amylase and α-glucosidase in vitro, with reported half-maximal inhibitory concentrations of 7.00 ± 0.29 and 8.11 ± 0.36 mg/mL, respectively [68]. Whether comparable inhibition occurs after oral consumption remains unknown.

A tocopherol-rich fraction from *C. pepo* seeds lowered glucose and improved selected insulin- and lipid-related variables in a poloxamer-407-induced rat model of type 2 diabetes, while molecular docking suggested potential interactions with targets regulating insulin signaling, glucose and lipid metabolism, and incretin activity [69]. The in vivo result and the computational predictions should be interpreted separately: neither demonstrates that these targets mediate a clinical effect. Reports of β-cell regeneration or restoration after pumpkin polysaccharides are likewise confined largely to chemically induced rodent models, and polysaccharides from different botanical species or preparations should not be treated as the same intervention [94].

Human evidence for pumpkin seeds remains preliminary. In a randomized, single-blind crossover trial, 15 normoglycemic adults consumed matched high-carbohydrate meals containing no seed, 65 g of pumpkin seeds, or 65 g of flaxseeds. Addition of pumpkin seeds reduced the two-hour incremental glucose area under the curve by approximately 35% relative to the control meal [95]. Because this was a single-meal response in healthy adults and used a relatively large seed portion, it does not show sustained glycemic control, improved HbA1c or treatment of type 2 diabetes. Long-term trials using characterized preparations and clinically relevant endpoints remain necessary.

At the evidence-synthesis level, a 2026 systematic review identified nine controlled clinical trials involving 316 adults with or without type 2 diabetes [11]. The included studies differed substantially in species, anatomical fraction, preparation, dose, intervention duration, participant characteristics, and glycemic outcomes. The review therefore characterized the clinical evidence as inconsistent and concluded that additional high-quality, long-term trials are required before firm conclusions can be drawn.

Overall, fruit, pulp, polysaccharide, and seed preparations have produced glycemic signals across experimental and limited human studies. Nevertheless, differences in species, anatomical fraction, preparation, dose, study population, and outcome prevent these findings from being combined into a general antidiabetic claim for pumpkin. Longer-term randomized trials using botanically authenticated and compositionally characterized preparations are required before therapeutic efficacy can be established.

### 6.3. Antioxidant and Anti-Inflammatory Responses

Carotenoids, phenolics, and tocopherols can scavenge radicals or interrupt lipid oxidation in chemical and membrane systems, while unsaturated lipids and phytosterols may influence inflammatory and metabolic signaling [21]. These mechanisms make antioxidant and anti-inflammatory activity plausible. However, global results from standard in vitro assays—such as DPPH, 2,2′-azino-bis(3-ethylbenzothiazoline-6-sulfonic acid) (ABTS), and FRAP—or general total-antioxidant-capacity metrics should not be described as protection from disease [91]. Such assays depend strongly on extraction solvent, concentration, and reaction chemistry, and they do not reproduce physiological absorption or metabolism.

In *C. pepo* seeds, γ-tocopherol is often the predominant tocopherol, although its concentration and the relative proportions of tocopherol isomers vary among cultivars [23,27].

In vivo evidence is mainly preclinical. In adjuvant-arthritis rats, oils from Egyptian *C. moschata* and European *C. pepo* seeds reduced paw edema and altered tumor necrosis factor alpha (TNF-α), erythrocyte sedimentation rate and oxidative-stress markers under the tested conditions [70]. This supports activity in that model but does not establish treatment of human arthritis or synergy with standard drugs. More broadly, increases in superoxide dismutase, catalase or glutathione-related measures and decreases in malondialdehyde should be described as biomarker changes; their clinical relevance depends on the model, dose, assay and relation to functional outcomes. Limited human evidence also includes an eight-week randomized trial of *C. ficifolia* in adults with type 2 diabetes that assessed high-sensitivity C-reactive protein [66]. These findings concern an inflammatory biomarker and do not establish efficacy for treating inflammatory disease.

### 6.4. Antimicrobial and Antiparasitic Activity

Extracts and isolated seed proteins have inhibited selected bacteria, yeasts or filamentous fungi in vitro [71]. These observations are compatible with membrane disruption, enzyme inhibition or interference with protein synthesis, but activity at high microgram- or milligram-per-milliliter concentrations should not be labeled broadly “potent.” Minimum inhibitory or bactericidal values are specific to the organism, extract and protocol, and comparison with an antibiotic requires matched assay conditions. At present, pumpkin preparations have not been shown in controlled human trials to treat bacterial, fungal or viral infection.

The traditional anthelmintic use of pumpkin seed has a more direct, though still limited, evidence base. Extracts of *C. pepo* seeds have shown activity against selected helminths in vitro and in animal models, with the magnitude of the effect varying according to the extraction solvent and chemical profile [72]. In a community-based study of human taeniasis, an intestinal tapeworm infection, pumpkin seed combined with areca-nut extract produced an 89% expulsion rate and acted faster than either preparation alone [73]. The result applies to the combination, not pumpkin monotherapy; transient nausea and dizziness were also common. It therefore does not justify describing pumpkin seed as a clinically established broad-spectrum antiparasitic treatment.

### 6.5. Prostate and Lower Urinary Tract Symptoms

LUTS are the domain with the most substantial human evidence, although results remain mixed. Proposed mechanisms include inhibition of 5α-reductase by lipid-soluble constituents, modulation of androgen signaling, anti-inflammatory effects and changes in bladder function [14]. Pumpkin seed oil reduced testosterone-induced prostate enlargement in rats, but a mechanistic pathway demonstrated in vitro or in animals should not be presented as the confirmed cause of symptom change in patients. Symptom relief also does not imply reduction in prostate volume.

In the 12-month German Research Activities on Natural Urologicals (GRANU) study, 1431 men with benign prostatic hyperplasia (BPH)-associated LUTS were randomized to whole pumpkin seed (5 g twice daily), pumpkin seed extract (500 mg twice daily), or placebo. Response was defined as a reduction of ≥5 points in the International Prostate Symptom Score (IPSS) after 12 months. In the intention-to-treat analysis using last observation carried forward, the reported odds ratio for response was 0.65 (95% CI 0.50–0.84) for placebo versus whole seed, favoring whole seed, and 1.06 (95% CI 0.82–1.37) for placebo versus extract. The whole-seed comparison was interpreted descriptively because the preceding extract–placebo comparison was not significant under the prespecified hierarchical testing procedure; the whole-seed arm could not be blinded, and the findings require confirmation [96]. In a smaller 12-month trial comparing pumpkin seed oil, saw palmetto oil, their combination, and placebo, mean IPSS in the pumpkin seed oil group decreased from 20.7 to 8.7, representing a within-group reduction of 12.0 points. However, between-group differences were not statistically significant, only 47 men completed the study, and prostate volume did not change significantly [74]. In a 2021 single-blind comparison, the reported mean within-group IPSS changes over three months were −3.19 points (95% CI −4.40 to −1.98) with pumpkin seed oil and −5.33 points (95% CI −6.69 to −3.97) with tamsulosin. The between-group comparison of changes favored tamsulosin (*p* = 0.020), but the study had no placebo arm [75].

Evidence for overactive bladder is weaker. In 45 volunteers given 10 g/day *C. maxima* seed oil for 12 weeks, Overactive Bladder Symptom Scores decreased from baseline [76]. Because this was an uncontrolled study based on self-reported symptoms, placebo effects, regression to the mean and natural variation cannot be excluded.

The 2013 European Medicines Agency monograph should be represented precisely. It recognizes the traditional use of *C. pepo* seed preparations for the relief of LUTS related to benign prostatic hyperplasia or overactive bladder after serious conditions have been excluded by a physician; it is not an approval based on well-established clinical efficacy and does not claim that the preparation reduces prostate enlargement. A later 2022 addendum concluded that newly identified small studies did not justify revising the monograph [7]. Persistent, worsening, or alarm symptoms require medical evaluation rather than self-treatment.

### 6.6. Antiproliferative and Chemopreventive Observations

Anticancer claims require especially cautious wording. A hydroethanolic extract of Styrian *C. pepo* seed reduced viability by approximately 40–50% in several hyperplastic and cancer cell lines under the tested conditions; a non-hyperplastic fibroblast line also showed about 20% growth inhibition [77]. Other solvent fractions have altered proliferation, cell-cycle distribution or autophagy in PC-3 prostate cancer cells [78]. These experiments identify leads for fractionation and mechanistic work, but they do not establish selectivity, bioavailability, an effective human dose or tumor control in vivo.

Cucurbitacins isolated from wild or non-food *Cucurbita* material can disrupt cytoskeletal or signaling pathways in cultured tumor cells, but purified compounds cannot be generalized to edible pumpkin flesh, seed or oil [79]. Concentrations that produce cytotoxicity in vitro may be pharmacologically or toxicologically irrelevant after dietary exposure. Taken together, these in vitro findings warrant further investigation but do not establish the efficacy of *Cucurbita* preparations for cancer prevention or treatment in humans.

### 6.7. Neurobehavioral, Hepatic, and Hematological Outcomes

Pumpkin seeds provide protein, magnesium, zinc, and tryptophan [22]; however, nutrient composition alone does not establish antidepressant or neuroprotective effects. The Antidepressant Food Score is a nutrient-profiling system intended to inform dietary research and recommendations; this classification does not, by itself, establish the antidepressant efficacy of pumpkin or pumpkin seeds [97]. In mice, oral administration of a baked *C. moschata* preparation (Sweetme Sweet Pumpkin™, DERGOUN Co., Iksan, Republic of Korea) for 28 days reduced immobility time in the forced swimming test [98]. These findings are limited to a preclinical model and do not establish antidepressant efficacy in humans. Human evidence includes a seven-person randomized crossover pilot in which deoiled gourd-seed protein with carbohydrate was associated with improvement in one objective anxiety measure [87]. A separate study in 40 young women assessed memory outcomes after a mixture of pumpkin seed, sunflower seed, and black mulberry, with or without exercise, preventing attribution to pumpkin seed alone [88].

Hepatic and lipid effects are also predominantly preclinical. In an atherogenic mouse model, replacing dietary saturated fat with polyunsaturated-fat-rich pumpkin seed oil attenuated selected measures of steatosis and atherosclerosis, and virgin oil produced additional effects relative to refined oil [80]. The study supports a diet-composition hypothesis in that model; it does not show that pumpkin seed oil restores human liver function or treats nonalcoholic fatty liver disease. Changes in alanine aminotransferase (ALT), aspartate aminotransferase (AST), lipids or histology should be described individually, and studies using mixtures with flaxseed or other foods cannot isolate a pumpkin-specific effect. Human lipid evidence includes a 12-week randomized pilot trial in 35 postmenopausal women comparing pumpkin seed oil (2 g/day) with wheat germ oil. HDL cholesterol increased within the pumpkin seed oil group, but this preliminary biomarker finding does not establish cardiovascular benefit [84].

Pumpkin kernels contain non-heme iron, but clinical evidence for anemia prevention is insufficient [99]. Eight healthy women consumed 30 g/day pumpkin seed kernels together with 30 g/day iron-fortified cereal for four weeks. Serum iron and transferrin saturation increased and total iron-binding capacity decreased, while hemoglobin and ferritin did not establish correction of anemia [81]. The very small uncontrolled pre–post design, healthy population and combined intervention preclude attribution to pumpkin seed or a conclusion about treatment of iron-deficiency anemia. In a six-month cluster-randomized study in preschool children, *C. moschata* seed paste was compared with pumpkin flesh, and improvements in selected iron-status biomarkers were reported [85]. Inconsistent reporting of between-group effects and the absence of hemoglobin measurements limit conclusions about anemia prevention or treatment. Table 5 complements the evidence overview in Figure 3 by summarizing representative preparations and interventions, the highest level of direct evidence, principal findings, and limitations relevant to their interpretation.

### 6.8. Integrated Assessment and Priorities for Translation

The biological evidence is uneven across outcomes. Human studies are most developed for LUTS, although findings differ among whole seeds, extracts and oils. A patient-level pooled analysis reported an adjusted 0.7-point International Prostate Symptom Score (IPSS) difference (95% CI 0.1–1.2) favoring a proprietary soft extract, although the two component trials yielded contradictory findings and the analysis was funded by the product manufacturer [10]. A small controlled crossover trial also reported reduced postprandial glycemia after a meal containing pumpkin seeds [95]. Evidence for antimicrobial, antiparasitic, anticancer, neurobehavioral, hepatic and anemia-related effects remains predominantly preclinical or derives from limited, indirect or multicomponent human studies.

Clinical development should begin with a defined intervention, characterized according to Section 3.6, and a dose appropriate to the intended food, supplement or pharmacological use. Whole foods, crude extracts and purified constituents require separate evaluation because their composition and achievable exposure differ. Mechanistic findings can guide outcome selection, while studies of digestion, absorption and metabolism should help establish exposure at the relevant site of action. This is particularly important when experimental activity has been observed only at concentrations whose relevance to human consumption is uncertain.

Trials should preregister clinically meaningful primary outcomes, justify sample size, use credible comparators and report adherence, diet, concomitant treatment, adverse events and null findings. Glycemic studies should assess sustained effects using fasting glucose, HbA1c or continuous-glucose measures in an appropriate population. Urinary studies should combine validated symptom scores with objective flow or residual-volume measures, suitable blinding and adequate follow-up. For less developed indications, pharmacokinetic and toxicological evaluation should guide progression to efficacy trials. Prospective safety monitoring should address tolerability and potential interactions, particularly for concentrated preparations. Independent replication is needed to determine whether observed benefits are reproducible for the specified preparation and use.

## 7. Modern Applications and Technology Readiness

Modern applications of *Cucurbita* and its by-products can be grouped into food ingredients with demonstrated techno-functional effects, nutraceutical and delivery systems evaluated mainly at laboratory scale, active packaging materials, and cosmetic or biomedical candidates that remain exploratory. Here, techno-functional properties refer to ingredient characteristics that affect food manufacture and structure, including water binding, emulsification, viscosity, and texture. A recent comprehensive review maps this broad field [5], but these categories should not be presented as equally mature. Product performance depends on the *Cucurbita* species and cultivar, anatomical fraction, pretreatment, inclusion level, carrier composition, processing conditions, and storage environment. Accordingly, changes in composition, chemical antioxidant capacity, or in vitro bioaccessibility should not automatically be interpreted as evidence of improved human health.

### 7.1. Food Ingredients and Formulated Foods

#### 7.1.1. Conversion of By-Products into Functional Ingredients

Peels, fibrous pulp, seed-use residues, and press cakes can be converted into powders, flours, protein-rich fractions, and oil ingredients through drying and fractionation. These operations improve stability and facilitate incorporation into formulated foods, but their effects are strongly process-dependent [22]. In the flesh of seed-type pumpkins, far-infrared drying produced the highest measured polyphenol content, whereas freeze-drying preserved β-carotene and ascorbic acid better than hot-air drying under the tested conditions [62]. In a separate study, foam-mat drying, in which a stabilized whipped purée is dried as a thin porous layer, increased the drying rate of pumpkin purée relative to conventional convective drying at the same temperature, and the flour produced at 45 °C retained carotenoids and exhibited high water-holding capacity [100]. Microwave-vacuum drying retained more total carotenoids than hot-air drying in pumpkin slices, although increasing microwave power promoted the loss and isomerization of all-trans carotenoids [101]. Process selection should therefore balance drying efficiency, ingredient functionality, sensory quality, and bioactive retention rather than assuming that one method is universally superior.

#### 7.1.2. Bakery, Cereal, and Snack Applications

Pumpkin seed flour provides protein, unsaturated lipids, and minerals, whereas peel, pomace, and pulp powders are principally used to increase dietary fiber, carotenoid content, and water-binding capacity. In bread and other cereal products, low-to-moderate substitution can improve nutritional composition, but increasing the proportion of pumpkin material frequently changes dough development, loaf volume, crumb color, hardness, and flavor [102]. Yellow-pumpkin powder at 5% did not compromise the overall acceptability of pan or pita bread, whereas higher substitution increased measured phenolic content but reduced pan-bread volume and specific volume [102].

Other primary formulation studies demonstrate similar matrix-specific trade-offs. Incorporating 10–25% pumpkin pomace powder increased dough water absorption, stability, and development time but reduced cookie spread, increased breaking strength, and progressively lowered overall acceptability [37]. Replacing wheat flour with pumpkin seed press-cake flour improved the calculated nutritional profile of muffins, but peroxide and anisidine values, indicators of primary and secondary lipid oxidation, respectively, increased with the proportion of pumpkin seed flour and storage time; a 33% formulation remained acceptable under the particular two-week storage conditions tested [103]. In gluten-free pasta, the formulation containing 25% pumpkin flour was the most desirable among the tested combinations, whereas higher replacement produced different textural and sensory effects [104]. These inclusion levels are formulation-specific findings rather than universal optima. Claims concerning immune enhancement, recovery from infection, or prevention of age-related disease cannot be inferred from nutrient enrichment or product acceptability alone.

#### 7.1.3. Meat, Dairy, Beverage, and Emulsion Systems

Pumpkin pulp, seed flour, and seed paste have been investigated as water-binding agents and partial replacements for meat or animal fat. In beef patties, replacing 2–5% of lean meat with a dried pumpkin-pulp and seed mixture increased water-holding capacity but did not significantly change cooking yield or diameter; sensory scores were also not significantly different under the conditions tested [105]. In beef meatballs, replacing fat with pumpkin seed-kernel flour reduced total fat and improved cooking loss and several fatty-acid indices, but higher inclusion reduced redness and sensory scores; the 3% formulation was closest to the control [106]. In a cooked meat-batter model, pumpkin seed paste used as a complete pork-backfat replacement increased the polyunsaturated-fatty-acid proportion and reduced lipid oxidation, although it also changed product color and texture [107]. These results demonstrate dose- and matrix-dependent ingredient functionality rather than direct health benefits.

Pumpkin ingredients have also been evaluated in fermented dairy systems. In stirred yogurt, adding 15% pumpkin pulp increased water-holding capacity and apparent viscosity, while lactic-acid-bacteria counts were not adversely affected during 14 days of refrigerated storage; sensory effects differed among the pumpkin varieties tested [108]. In beverages, HPH can improve the dispersion and transfer of carotenoids from pulp or pomace; in Hokkaido pumpkin purée, however, PEF did not produce the same improvement, illustrating matrix and process dependence [26]. In a separate batch-processing study of pumpkin juice and nectar inoculated with *Escherichia coli*, PEF achieved a maximum reduction of approximately 2.5–3 log_10_ at a specific energy of 80 J/g and electric field strengths above 26 kV/cm [109]. Nonthermal treatments should not be described as validated pasteurization replacements unless target organisms, treatment energy, temperature history, log-reduction values, and storage performance have been established for the final product.

### 7.2. Nutraceutical Products and Delivery Systems

#### 7.2.1. Seed Oil, Proteins, and Polysaccharides

Pumpkin seed oil is a commercially relevant food and supplement ingredient containing linoleic and oleic acids, phytosterols, squalene, and tocopherols [39]. Its composition varies with cultivar, cultivation conditions, roasting, pressing, and storage. Cold pressing may favor a minimally processed product, whereas thermal pretreatment can increase oil yield and alter flavor or the extractability of minor compounds; the preferred process therefore depends on the intended product and quality criteria [41]. Pumpkin seed oil should not be presented as nutritionally interchangeable with olive oil or other culinary oils without considering fatty-acid composition, oxidative stability, sensory use, and price.

Human evidence is available for selected standardized pumpkin seed preparations, particularly for LUTS associated with benign prostatic hyperplasia [10,74,75]. These findings should not be generalized to every cold-pressed oil, capsule, or seed extract.

Evidence concerning pumpkin polysaccharides remains preclinical and preparation-specific. A protein-bound polysaccharide isolated from pumpkin fruit reduced blood glucose and increased serum insulin in alloxan-induced diabetic rats [110]. Another pumpkin-polysaccharide preparation affected glycemic and lipid endpoints and altered the gut microbiota in a high-fat-diet rat model of type 2 diabetes [111]. An acidic polysaccharide isolated from *C. moschata* also showed cytoprotective and antioxidant effects in hydrogen-peroxide-stressed mouse macrophages [112]. These animal and cell-culture findings do not establish hypoglycemic, hepatoprotective, or antioxidant efficacy in humans, and the different preparations should not be treated as compositionally equivalent.

#### 7.2.2. Encapsulation and Controlled Release

Encapsulation is being investigated to protect carotenoids, polyunsaturated lipids, and protein hydrolysates from oxidation and gastrointestinal degradation. Alginate- and chitosan-based microgels can reduce the initial release of pumpkin seed protein hydrolysates and modify peptide diffusion during simulated gastrointestinal digestion [113]. These systems provide proof of controlled release, but simulated digestion does not demonstrate intestinal absorption or clinical efficacy. Similarly, nanostructured lipid carriers can increase the in vitro bioaccessibility of carotenoid-rich extracts, while carbohydrate- or starch-based microencapsulation can improve the oxidative stability of pumpkin seed oil during storage [114]. Carrier composition, loading efficiency, water activity, storage temperature, and compatibility with the final food matrix remain central design variables.

A recent formulation coupled SC–CO_2_ extraction of lutein-rich pumpkin peel extract with food-grade nanoliposomes. In the best-performing formulation, approximately 73% of lutein was released and 45.2% became micellarized during static in vitro digestion [115]. These results demonstrate improved release and micellar bioaccessibility within that formulation; they do not establish human absorption, tissue delivery, or therapeutic bioavailability. In vivo pharmacokinetic studies, dose-standardization, long-term stability testing, and evaluation in real foods are needed before clinical or commercial conclusions can be drawn.

### 7.3. Active and Bio-Based Packaging for Food Preservation

Active packaging—packaging designed to interact with the food or package environment to preserve quality or extend shelf life—provides another route for valorizing pumpkin residues. Relevant approaches include active edible coatings as well as bio-based films that provide primarily passive barrier functions. Chitosan coatings used together with vacuum packaging and refrigerated storage have been investigated for maintaining the firmness, color, β-carotene content, and microbiological quality of fresh-cut pumpkin [116]. Pumpkin seed oil cake has also been incorporated into a proteinaceous layer laminated with polyethylene, producing a prototype material with useful oxygen- and water-vapor-barrier behavior under the tested conditions [38]. Laboratory films cannot yet be considered direct replacements for conventional plastics. Commercial assessment requires mechanical testing under realistic humidity, sealability, migration and allergenicity testing, sensory evaluation, biodegradation under defined conditions, and techno-economic comparison with established packaging.

Peel and pulp extracts may provide antioxidant or antimicrobial activity in coatings, but efficacy must be confirmed in the target food rather than inferred from agar-diffusion or chemical assays. Extract-mediated synthesis of metallic nanoparticles is also exploratory: composition, dose, migration, cytotoxicity, environmental persistence, and regulatory status must be established before food or biomedical use. These requirements are more informative than the broad label “green synthesis” alone.

### 7.4. Cosmetic and Topical Applications

Pumpkin peel, pulp, and seed oil are potential sources of ingredients for cosmetic formulations, but most evidence remains in vitro or preclinical. Extracts prepared using water, aqueous propylene glycol, or ethanol differ substantially in phenolic composition and activity according to solvent, species, and cultivar [117]. In a study of peel extracts from five *Cucurbita maxima* and *C. moschata* cultivars, the investigators measured chemical antioxidant activity, tyrosinase inhibition, in vitro sun-protection factor, and keratinocyte viability; the extracts were non-cytotoxic in the tested cell model at concentrations up to 1000 μg/mL [36]. These outcomes support further formulation research, but they do not establish clinical sunscreen efficacy, absence of irritation or sensitization, or effectiveness against dermatological disease.

Pumpkin seed oil can function as an emollient lipid because of its fatty-acid and tocopherol content [39]. More specific claims concerning collagen stimulation, sebaceous regulation, acne, psoriasis, hyperpigmentation, wrinkle reduction, or hair repair require direct topical studies. Oral pumpkin seed oil was evaluated in one randomized trial of men with androgenetic alopecia, but the result concerns a specific oral capsule and should not be transferred to topical cosmetics [118]. Nanoemulsions, liposomes, and nanostructured lipid carriers may improve ingredient stability and deposition, but final products still require validated irritation, sensitization, phototoxicity, microbiological, and stability testing [36].

### 7.5. Application Readiness, Safety, and Commercialization

The most immediate opportunities for *Cucurbita* by-products are food ingredients whose compositional and technological benefits can be measured in formulated products, including protein- and fiber-rich flours, oils and pigment-containing powders. Relevant performance measures include water binding, emulsion behavior, pigment delivery and oxidative stability. Selected packaging materials also show promise, while encapsulation systems, topical extracts and green-synthesized nanoparticles require further development in realistic product matrices.

Translation requires comparison with established ingredients, sensory acceptance at the proposed inclusion level, validated shelf life and reproducible pilot-scale production. Safety assessment should build on the quality controls outlined in Section 3.6 and address the intended exposure route. For concentrated oral preparations, cucurbitacin exposure, allergenicity and antinutritional factors warrant particular attention. Primary research has identified 2S albumins and 11S globulins as allergens in pumpkin seeds, and seed-induced anaphylaxis has been documented in a child who tolerated pumpkin pulp [119,120]. These findings indicate that tolerance to pulp cannot be assumed to establish tolerance to seed-derived preparations.

Contaminant occurrence further supports the need for analytical quality controls. An Iranian survey reported lead and cadmium contamination in pumpkin seed samples, while a survey in southern Punjab, Pakistan, detected aflatoxins in pumpkin seeds sold with and without shells [121,122]. In an experiment using historically contaminated soils, mean dieldrin concentrations in *C. pepo* fruits varied among cultivars from 4.9 ± 1.1 to 70.3 ± 3.6 μg/kg fresh weight [123]. These findings support screening strategies that consider raw material origin, cultivation conditions and storage practices. Topical products require irritation and sensitization testing. For packaging and nanoparticle applications, migration and ecotoxicity should be assessed where relevant to the intended use.

Regulatory classification should be established for the intended use and claims, since food, supplement, cosmetic, medical-device and pharmaceutical products follow different evidentiary and regulatory pathways. In the European Union, preparations meeting the definition of a novel food require authorization under Regulation (EU) 2015/2283 [17]. EFSA’s updated guidance specifies the scientific information required for safety assessment, including the production process, composition, proposed uses and intake, toxicological data and allergenicity [16]. The regulatory status of a specific extract or preparation therefore requires consideration of its composition, production process and history of consumption. Nutrition and health claims are governed separately by Regulation (EC) No 1924/2006 [15]; evidence supporting ingredient safety does not by itself substantiate a claimed health benefit.

Commercial feasibility should be evaluated alongside the process economics and environmental criteria outlined in Section 4.7, with attention to production costs and achievable product performance. Table 6 compares the demonstrated outcomes, evidence level, technological readiness and principal limitations of current and emerging applications.

## 8. Conclusions and Future Perspectives

*Cucurbita* biomass provides complementary resources for food ingredients and functional materials. Pulp supplies carotenoids and polysaccharides; seeds provide protein, while seed oil supplies unsaturated lipids, tocopherols, phytosterols and squalene; and peels, pomace, seed coats and press cakes retain pigments, phenolics, fiber and other recoverable constituents. This diversity supports integrated use of the fruit and its processing by-products, with each fraction directed toward applications suited to its composition.

A central conclusion is that measured concentration, true retention, analytical extractability, digestive bioaccessibility and human bioavailability describe different outcomes. Maintaining these distinctions improves the interpretation of processing studies and helps identify which changes are relevant to a final product. Likewise, biological conclusions depend on the species, fraction, preparation and experimental level actually investigated. The resulting framework links material identity and chemical characterization to processing performance, biological evidence and application readiness.

The clearest near-term opportunities concern ingredients with measurable nutritional or technological functions. Clinical translation is more limited: selected seed preparations have the most developed human evidence for LUTS, with variable results, while many other proposed health applications remain experimental. Development priorities should therefore reflect both the intended use and the strength of the evidence supporting the specific preparation.

Future research would benefit from coordinated studies that follow the same characterized material through extraction, processing, formulation and evaluation. Such studies could connect compositional changes with product performance and identify markers useful for batch consistency. Shared reference materials and interlaboratory comparisons would strengthen the comparability of results. At pilot scale, integrated recovery routes should be assessed for their capacity to produce several usable fractions with acceptable resource demand and cost. For health applications, exposure and clinical studies should evaluate the preparation intended for eventual use. Linking these stages would help establish which valorization routes can deliver reproducible products with demonstrable functionality, safety and environmental value.

## Figures and Tables

**Table 1:** Fraction-specific composition, key analytical considerations, and potential valorization routes for *Cucurbita* biomass.

Fraction	Principal Constituents	Representative Compositional Features	Key Characterization, Reporting, and Quality Markers	Potential Valorization Routes	References
Pulp/flesh	Carotenoids; sugars; soluble and structural polysaccharides; minerals	α- and β-carotene, lutein, zeaxanthin, violaxanthin, and carotenoid esters; composition is strongly cultivar- and maturity-dependent	HPLC-DAD or LC-MS carotenoid profile; fresh- or dry-mass reporting basis and true retention; monosaccharide composition and molecular-mass distribution of polysaccharides; degree of esterification for pectin	Purées, beverages, powders, natural colorants, fiber-rich ingredients, and carotenoid delivery systems	[5,9,18,19,20]
Peel/rind	Carotenoids; phenolic compounds; cellulose, hemicellulose, and pectin	Pigments and phenolics may be more concentrated per gram than in pulp, but tissue depth and fresh- versus dry-mass basis materially affect comparisons	HPLC-DAD or LC-MS carotenoid profile; chromatographic phenolic profile; fiber fractions; cucurbitacin screening when bitterness or atypical botanical material is present	Pigment and phenolic extracts, fiber or pectin ingredients, topical formulations, and active-packaging components	[9,20,21,22]
Seeds/kernels	Proteins; lipids; dietary fiber; phenolic compounds; minerals; cucurbitine	Approximately 14–38% protein and 22–55% lipid have been reported, depending on species, dehulling, maturity, and reporting basis; linoleic and oleic acids generally predominate in the oil fraction	Proximate composition; amino-acid and protein/peptide profiles; GC-FID fatty-acid profile; protein digestibility and functionality; allergenicity, phytate, oxalate, microbial quality, and contaminant testing	Flours, protein concentrates or isolates, snacks, nutraceutical preparations, and substrates for oil recovery	[9,19,21,22]
Seed oil	Triacylglycerols; tocopherols; phytosterols; squalene; minor carotenoids and phenolic compounds	Unsaturated fatty acids commonly exceed 70%; tocopherol and Δ^7^-sterol profiles vary with genotype and processing	GC-FID fatty-acid and sterol profiles; HPLC tocopherols; peroxide value, *p*-anisidine value, acid value, and oxidative-stability indices; residual-solvent and authenticity testing	Edible oil, standardized lipid extracts or capsules, lipid carriers, and cosmetic emollient formulations	[23]
Fruit pomace	Dietary fiber; residual carotenoids and phenolic compounds; variable amounts of seed-derived protein and lipid	Composition depends on the proportions of residual pulp, peel, and seeds and on processing and juice-clarification efficiency	Tissue composition; particle size; moisture and water activity; carotenoid and phenolic profiles; microbial quality; batch-to-batch fingerprint	Fiber-rich food ingredients, secondary pigment or phenolic extraction, feed, films, and feedstock for sequential recovery of multiple products	[5,24]
Seed press cake/oil cake	Proteins; dietary fiber; residual oil; phenolic compounds; minerals	Composition depends on dehulling, thermal pretreatment, pressing efficiency, and subsequent defatting	Proximate composition; residual oil and oxidation status; amino-acid profile; protein digestibility and techno-functional properties; phytate; allergenicity, microbial quality, and contaminants	Flours, protein concentrates or isolates, secondary oil recovery, feed, films, and other protein-based materials	[24]
Other tissues: flowers, leaves, and germinating seeds	Polyphenols, carotenoids, proteins, enzymes, and other specialized metabolites	Promising but less comprehensively characterized than fruit and seed fractions	Botanical authentication; targeted and untargeted LC-MS; proteomic or enzyme assays; safety and contaminant assessment	Specialty foods, enzyme or protein discovery, and early-stage bioactive research	[6]

Values and ranges are representative of the cited studies and may vary with species, cultivar, anatomical fraction, maturity, processing, analytical method, dehulling, and fresh- or dry-mass reporting basis. Water activity refers to the availability of water for microbial growth and chemical reactions and is distinct from total moisture content. Abbreviations: GC-FID, gas chromatography with flame-ionization detection; HPLC, high-performance liquid chromatography; HPLC-DAD, high-performance liquid chromatography with diode-array detection; LC-MS, liquid chromatography–mass spectrometry.

**Table 2:** Comparative assessment of extraction technologies applicable to *Cucurbita* matrices.

Approach	Principal Targets and Matrices	Principal Advantages	Main Limitations and Development Status	References
Conventional solvent extraction and mechanical pressing	Phenolic compounds extracted using aqueous alcohols; oils and other lipophilic compounds extracted using non-polar solvents; seed oil recovered by mechanical pressing	Established methods; relatively simple equipment; useful reference procedures; solvent extraction can provide high oil recovery; mechanical pressing avoids extraction solvents	Solvent extraction can require long processing or solvent-recovery steps and creates residue- and waste-management requirements; exposure to heat and oxygen may affect labile constituents. Pressing efficiency depends on seed moisture and pretreatment and may leave appreciable residual oil. Industrially established.	[26,39]
SC–CO_2_ extraction	Seed lipids, phytosterols, tocopherols and other lipophilic constituents	Moderate operating temperatures; tunable solvent density and selectivity; extract separation by depressurization; no residual organic extraction solvent when no modifier is used	High capital cost and energy demand for CO_2_ compression and recirculation; limited recovery of polar compounds; a co-solvent and additional purification may be required; oil yield may be lower than with solvent extraction. The platform is industrially established for selected high-value extracts, but *Cucurbita*-specific evidence remains predominantly laboratory-scale.	[34,40,41]
DES/NADES	Hydrophilic systems for phenolic compounds; hydrophobic systems for carotenoids and other lipophilic compounds from peel and pulp	Adjustable polarity; very low volatility; compatibility with UAE; formulation can be tailored to the target compound	High viscosity and slow mass transfer; difficult target–solvent separation and solvent recycling; formulation-specific toxicity, biodegradability, residue, sensory, and regulatory questions. Predominantly laboratory-scale for *Cucurbita*.	[42,43,44]
EAAE	Seed-oil and protein fractions; carotenoids and other intracellular constituents from seed, peel, or pulp matrices	Water-based processing under mild conditions; possibility of simultaneous oil and protein recovery; reduced use of organic solvents	Enzyme cost and processing time; stable-emulsion formation; complex phase separation; wastewater and enzyme-reuse considerations; compositional and stability gains are not uniform. Predominantly laboratory-scale for *Cucurbita*.	[35,45]
UAE	Phenolics, carotenoids and lipids from comminuted peel, pulp, pomace and seeds	Rapid mass transfer; shorter treatment times than conventional maceration; compatibility with ethanol, water, edible oils, or DES	Non-uniform cavitation during scale-up; possible oxidation and, in probe systems, probe erosion; performance depends on acoustic power density, equipment geometry, and temperature control. Predominantly laboratory-scale for *Cucurbita*; pilot and continuous configurations require process-specific validation.	[13,46,47]
MAE	Phenolic compounds and pigments in matrices and solvents that efficiently absorb microwave energy	Rapid volumetric heating; short extraction times; reduced solvent viscosity and faster solubilization	Hot spots and localized degradation; limited penetration into large material beds; weak coupling with non-polar media; increasingly difficult pressure and temperature control during scale-up. Predominantly laboratory-scale for *Cucurbita*.	[13,46,47]
HPH, PEF, and hybrid pretreatments	Carotenoid transfer from purée to juice; cell disruption before aqueous or solvent extraction; combined recovery of multiple fractions	HPH can markedly increase tissue disruption and carotenoid transfer; enzymatic and mechanical treatments may be complementary; individual unit operations can be integrated into continuous processing	Additional equipment and process-control requirements; HPH can generate heat and consume substantial energy; PEF response is strongly matrix-dependent and did not improve carotenoid transfer in the cited Hokkaido-pumpkin study; hybrid benefits must justify the additional complexity. Laboratory validation is available for *Cucurbita*, but broader scale-up requires process-specific confirmation	[19,26,35]

Development status refers primarily to evidence demonstrated directly in *Cucurbita* matrices; the same unit operations may be more technologically mature in other food and plant-processing systems. Abbreviations: DES, deep eutectic solvent; EAAE, enzyme-assisted aqueous extraction; HPH, high-pressure homogenization; MAE, microwave-assisted extraction; NADES, natural deep eutectic solvent; PEF, pulsed electric field; SC–CO_2_, supercritical carbon dioxide; UAE, ultrasound-assisted extraction.

**Table 3:** Representative extraction conditions and quantitative outcomes reported for *Cucurbita* matrices.

Matrix	Extraction Method and Solvent	Solvent-to-Solid Ratio	Temperature	Time and Additional Conditions	Reported Yield, Recovery, or Concentration	Reference
*C. pepo*, dried ground seeds	UAE; *n*-hexane	10 mL/g	22 °C	15 min	Oil yield: 24.6% of dry seed mass	[41]
*C. pepo*, ground seeds	SC–CO_2_	NR; CO_2_ flow: 120 g/min	40 °C	16 h; 30 MPa	Combined oil yield: 18.8% of seed mass, summed across three separator fractions	[41]
*C. pepo* cv. Herakles, hulless seeds	EAAE; water with a 2% enzyme cocktail	1 g water/g seeds	54 °C	15.4 h; pH 4.7	Oil recovery: 72.64 ± 0.61% of total seed oil; oil yield approximately 36.0% of dry seed mass	[35]
*C. maxima* cv. Gold Nugget, freeze-dried peel	UAE; corn oil	10 mL/g	22–25 °C	30 min sonication; 45 min elapsed time; 20% amplitude	Total carotenoid concentration: 38.03 ± 4.21 μg/g oil extract	[13]
*C. maxima* cv. Gold Nugget, freeze-dried peel	MAE; corn oil	10 mL/g	≤45 °C	30 min; 130 W; intermittent cooling	Total carotenoid concentration: 34.94 ± 3.60 μg/g oil extract	[13]
Pumpkin peel, freeze-dried; species NR	NaHDES screening; DL-menthol/lactic acid, 1:2 molar ratio	50 mL/g	25 ± 2 °C	30 min mixing followed by 60 min sonication; 45 kHz	Total carotenoid recovery: 25% of the exhaustive acetone-extraction benchmark	[44]
*C. pepo*, milled seed hulls	Stirred extraction; water	7.5 mL/g	60 °C	6 h; 300 rpm	Extract yield: 4.8 ± 0.2% of starting hull mass	[49]
*C. pepo*, milled seed hulls	Stirred extraction; ethanol/water, 1:1	5 mL/g	60 °C	6 h; 300 rpm	Extract yield: 4.2 ± 0.3% of starting hull mass	[49]

Abbreviations: UAE, ultrasound-assisted extraction; MAE, microwave-assisted extraction; EAAE, enzyme-assisted aqueous extraction; SC–CO_2_, supercritical carbon dioxide extraction; NaHDES, natural hydrophobic deep eutectic solvent; NR, not reported. Solvent-to-solid ratios retain the volume or mass basis reported in each study. Mass yield expresses recovered oil or extract relative to starting biomass; recovery expresses the fraction of the available target recovered. Carotenoid concentrations in corn oil refer to the final oil extract. These outcomes have different denominators and do not support direct ranking across studies. The NaHDES entry describes the initial screening experiment, rather than the subsequent optimization protocol.

**Table 4:** Principal effects and trade-offs of processing operations applied to *Cucurbita* matrices.

Operation and Typical Matrix	Dominant Compositional or Structural Effects	Potential Advantages	Main Limitations and Interpretive Cautions	References
Peeling, cutting, pureeing and juicing (flesh, peel, juice and pomace)	Removes or redistributes tissues; reduces particle size; releases cellular contents; increases contact with oxygen and endogenous enzymes	Improved dispersibility and extractability; softer texture; formation of juice and puree streams	Depletion of peel-associated constituents and insoluble fiber in peeled or clarified products; oxidation and enzymatic browning; transfer of constituents to pomace should not be interpreted as chemical degradation	[22]
Blanching and freezing (pieces or puree)	Enzyme inactivation and possible leaching during blanching; ice-crystal disruption and drip formation during freezing and thawing	Improved frozen-storage stability when blanching adequately controls endogenous enzymes	Heat-sensitive and water-soluble compounds can be lost; response depends on cultivar, piece size, blanching severity, freezing rate and storage	[51,52]
Controlled fermentation (juice, puree, flour or seed matrices)	Sugar consumption, acid production, phenolic biotransformation and modification of volatile and rheological profiles	Acidification, new sensory profiles and possible increases in extractable phenolics or strain-specific metabolites	Results are strain- and process-specific; viable LAB counts alone do not justify a probiotic claim; chemical antioxidant assays do not demonstrate in vivo benefit	[53,54,55]
Boiling, steaming and commercial thermal processing (pieces and puree)	Cell-wall softening, starch and pectin modification, enzyme and microbial inactivation, leaching, oxidation and carotenoid isomerization	Improved palatability and, under some conditions, carotenoid extractability or digestive bioaccessibility	Possible degradation or leaching of ascorbic acid and water-soluble phenolics; outcome depends strongly on cultivar, medium, geometry and time–temperature history	[12,51]
Roasting (seeds before consumption or oil pressing)	Protein denaturation, moisture loss, Maillard chemistry, volatile formation and possible lipid oxidation	Desirable roasted aroma; increased extractability and measured antioxidant response in some protocols	Excessive roasting may reduce protein quality and promote lipid-oxidation and other heat-generated products; study-specific optima should not be generalized	[58]
High-pressure-assisted thermal processing (puree)	Pressure- and temperature-dependent microbial and enzyme inactivation with disruption of cellular structures	Under the tested pressure–temperature combinations, short treatments maintained or increased analytically extractable carotenoids and phenolics and reduced PPO activity	Control of bacterial spores and shelf stability require separate validation; residual enzyme activity and storage temperature must also be considered	[60]
Hot-air, vacuum, infrared and freeze-drying (slices, pulp, peel, pomace and powders)	Water removal and concentration of solids; shrinkage or pore formation; thermal and oxidative reactions that vary with residence time and oxygen exposure	Reduced water activity and mass; powders with useful hydration properties; freeze-drying often retains color and labile compounds better than hot-air drying	Hot-air drying may cause shrinkage and thermal or oxidative losses, whereas freeze-drying is relatively slow and energy- and cost-intensive; comparisons require expression on a dry-matter basis or calculation of true retention, together with assessment during storage	[62]
Microwave-vacuum and multi-flash drying (slices)	Rapid internal heating and pressure-driven water removal create an expanded porous structure	Substantially shorter drying times and potentially rapid rehydration under the tested laboratory conditions	Scale-up, temperature uniformity, energy use and texture must be validated; rehydration performance depends on water temperature	[63]

Effects are matrix-, cultivar- and process-condition-dependent. Apparent increases in concentration may reflect water loss, tissue disruption or improved analytical extractability rather than net compound formation; expression on a dry-matter basis, true-retention calculations or mass-balance analysis should therefore be used where appropriate. Abbreviations: LAB, lactic acid bacteria; PPO, polyphenol oxidase.

**Table 5:** Evidence map of reported biological activities of *Cucurbita*-derived matrices.

Domain	Representative Matrix or Intervention	Highest Direct Evidence	Principal Observation	Evidence-Calibrated Interpretation	References
Glycemic control—fruit/pulp	Raw *C. ficifolia* fruit extract; 100 g/day of *C. ficifolia* fruit; lyophilized *C. maxima* pulp	Small eight-week randomized parallel-group trial in adults with type 2 diabetes; ten-person acute controlled study; uncontrolled 20-patient ICU before–after study	The eight-week *C. ficifolia* intervention reduced fasting glucose and HbA1c relative to dietary advice alone. A single dose of raw *C. ficifolia* extract reduced glucose over five hours. Mean glucose also decreased during three days of *C. maxima* pulp administration in critically ill diabetic patients, but insulin requirements did not decrease significantly.	Preliminary, preparation-specific evidence. Small groups, limited blinding, short follow-up, and the absence of a concurrent ICU control limit inference. Findings cannot be generalized across *Cucurbita* species or used to establish pumpkin pulp as a diabetes treatment or to claim human pancreatic β-cell regeneration.	[64,65,66,67]
Glycemic control—seeds	A mixed meal containing 65 g of pumpkin seeds; 10 g/day of pumpkin-seed powder	Small randomized acute crossover study in normoglycemic adults; open-label randomized controlled add-on study in adults with type 2 diabetes	The acute meal reduced 2-h postprandial glucose iAUC. In the six-month study, glycemic measures decreased within the seed-powder group, but unadjusted between-group comparisons at follow-up were not significant for fasting glucose, postprandial glucose, or HbA1c.	Preliminary acute and low-certainty longer-term evidence; insufficient to establish pumpkin seed as a diabetes treatment or to support claims of pancreatic β-cell restoration in humans.	[11,95]
Oxidative stress and inflammation	*C. ficifolia* fruit (100 g/day); pumpkin seed oil; sesame–pumpkin–flax mixture	Small eight-week randomized trial in adults with type 2 diabetes; single-arm human multiseed study; rat models	In the randomized trial, hsCRP decreased within the *C. ficifolia* group. The multiseed intervention was associated with changes in inflammatory markers, while pumpkin seed oils reduced paw edema and altered TNF-α and oxidative-stress biomarkers in rats.	Human findings concern inflammatory biomarkers and do not establish clinical benefit. The multiseed intervention prevents pumpkin-specific attribution. Evidence for oxidative-stress effects in the summarized studies remains preclinical.	[66,70,82]
Antimicrobial and antiparasitic activity	Pumpkin seed with areca-nut extract; pumpkin seed alone; isolated seed peptides and solvent extracts	Small nonrandomized community study for taeniasis; otherwise laboratory studies	Intact tapeworm expulsion occurred in approximately 89% of confirmed cases receiving the combination, versus 75% with pumpkin alone and 64% with areca alone. Cucurmoschin inhibited selected plant-pathogenic fungi in vitro.	Small nonrandomized groups and the combination treatment limit inference. No *Cucurbita*-derived preparation is an established stand-alone antibacterial, antifungal, antiviral, or anthelmintic treatment.	[73,83]
Prostate and urinary symptoms	Whole *Cucurbita pepo* seed; proprietary seed extract; seed oil	Large partially blinded randomized trial, pooled analysis of two placebo-controlled trials, and smaller active-comparator trials	In GRANU, the blinded extract comparison did not differ from placebo; the open whole-seed arm had a descriptively higher responder rate (58.5% versus 47.3%). Pooled extract results were small and inconsistent, and seed oil was less effective than tamsulosin.	The most clinically developed domain in the table, but preparations and findings are heterogeneous. Symptom improvement does not establish reduced prostate volume or disease modification.	[10,75,76,96]
Antiproliferative activity	Solvent-fractionated edible-seed extracts; separately, purified cucurbitacins from bitter *C. andreana* fruit	Cultured-cell studies	Seed extracts reduced viability or altered autophagy-related measures in selected cancer cell lines. Cucurbitacins isolated from *C. andreana* fruit inhibited selected cultured tumor cells.	Hypothesis-generating only. Purified compounds from bitter or wild fruit material cannot be generalized to edible pumpkin seed, flesh or oil; no human evidence supports cancer prevention or treatment.	[77,78,79]
Neurobehavioral outcomes	Deoiled gourd-seed meal plus carbohydrate; multicomponent seed/fruit formulations; seed extracts in rodents	Seven-person double-blind, placebo-controlled randomized crossover pilot; small multicomponent human study; otherwise animal evidence	The crossover pilot found improvement in one objective anxiety measure. A 40-person study used pumpkin seed together with black mulberry, sunflower seed and exercise, preventing pumpkin-specific attribution. Rodent studies report behavioral and biochemical changes.	Direct human evidence exists but is extremely limited and formulation-specific. These findings do not establish antidepressant, anxiolytic, cognitive-enhancing, or neuroprotective efficacy.	[87,88,89,97]
Circulating lipids	Pumpkin seed oil	Small randomized, double-blind pilot trial with wheat-germ oil as the comparator	In 35 postmenopausal women, 2 g/day for 12 weeks increased HDL cholesterol within the pumpkin-seed-oil group.	Preliminary biomarker evidence only; it does not establish lipid-lowering efficacy or reduction in cardiovascular events.	[84]
Hepatic and NAFLD outcomes	Virgin or refined seed oil used as dietary-fat replacement; pumpkin seed oil in high-fructose models	Mouse and rat studies	Model-specific improvements in steatosis, liver histology, inflammatory markers or atherosclerotic lesions have been reported.	No human evidence of NAFLD treatment. Dietary-fat substitution and model-specific designs limit attribution and do not demonstrate restored human liver function.	[80]
Iron status	100 g of *C. moschata* seed paste twice weekly; pumpkin kernels plus iron-fortified cereal	Six-month cluster-randomized preschool feeding study, with biochemical outcomes in 65 children; earlier uncontrolled eight-person study	The 2024 study reported favorable serum iron, ferritin and transferrin-saturation findings, although its results table and discussion are inconsistent about which effects differed between groups. Hemoglobin and anemia were not assessed. The earlier study combined kernels with fortified cereal.	Preliminary food-based nutritional evidence; insufficient to conclude that pumpkin seed alone prevents or treats iron-deficiency anemia.	[81,85]

The “highest direct evidence” column refers to the most informative evidence identified for the stated pumpkin preparation and outcome, not to the overall biological plausibility of the domain. Abbreviations: HbA1c, glycated hemoglobin; HDL, high-density lipoprotein; iAUC, incremental area under the curve; NAFLD, nonalcoholic fatty liver disease; TNF-α, tumor necrosis factor-α; ICU, intensive care unit; hsCRP, high-sensitivity C-reactive protein.

**Table 6:** Current and emerging applications of *Cucurbita*-derived ingredients and by-products, with evidence level and principal limitations.

Application	Representative *Cucurbita* Matrix or System	Directly Demonstrated Outcome	Evidence Level/Readiness	Principal Limitation	References
Bakery and cereal foods	Seed or press-cake flour; peel, pomace or pulp powder	Changes in nutrient composition, water absorption, dough rheology, product volume, color, texture and sensory acceptance	Laboratory formulation studies with product-level sensory testing	Higher inclusion can impair loaf volume, cookie geometry, texture, or sensory acceptance; acceptable levels are formulation-specific, and small sensory panels do not establish broad consumer acceptance	[37,102,103]
Meat and hybrid products	Seed-kernel flour; seed paste; dried pulp–seed mixture	Changes in water-holding and cooking properties, partial lean-meat or fat replacement, fatty-acid profile and lipid oxidation	Laboratory-scale prototype formulations with small sensory panels	Color, texture, and sensory effects depend on dose and matrix; long-term shelf-life validation and scale-up remain limited	[105,106,107]
Dairy and beverages	Pumpkin pulp in yogurt; pumpkin purée, juice and separated pomace	Pumpkin pulp increased yogurt water-holding capacity and viscosity without adversely affecting starter-culture counts under the tested storage conditions; HPH and enzyme treatment increased carotenoid transfer from purée to juice; PEF produced moderate reductions in inoculated *E. coli* under specified conditions	Laboratory formulation and processing studies	Effects are variety- and process-specific. The maximum reported PEF reduction was approximately 2.5–3 log for a single test organism under the tested conditions; this does not validate PEF as a stand-alone pasteurization process for pumpkin beverages	[26,108,109]
Oils, extracts, and prospective nutraceutical ingredients	Seed oil; defined whole-seed and proprietary seed-extract preparations; seed proteins and polysaccharide preparations	Nutritional and compositional value; limited human symptom evidence for defined whole-seed, oil, or extract preparations; protein and polysaccharide effects remain mainly preclinical	Marketed products exist, but clinical evidence ranges from limited human studies to preclinical research and varies markedly by preparation and indication	Whole seeds, oils, soft extracts, proteins, and polysaccharides are not interchangeable; efficacy is indication- and preparation-specific, and commercial availability does not establish clinical efficacy or regulatory approval	[10,111]
Encapsulated bioactives	Peel-derived carotenoids or lutein; pumpkin seed oil; seed protein hydrolysates	Formulation-dependent improvements in oxidative or storage stability and release during simulated digestion; increased micellar bioaccessibility in selected carotenoid systems	Laboratory proof of concept, including simulated gastrointestinal digestion	Simulated release and micellar bioaccessibility do not demonstrate human absorption, tissue delivery or clinical efficacy	[114,115]
Food-packaging materials	Pumpkin seed oil cake laminated to polyethylene	Water-vapor, oxygen, and light-barrier performance; mechanical characterization; proof-of-concept modified-atmosphere packaging	Laboratory-scale two-layer packaging prototype and model packing study	The laminate still contains polyethylene and retains some of the water sensitivity and mechanical limitations of its biopolymer layer. Food-contact migration, sealability, performance across relevant humidity conditions, end-of-life handling, and scale-up require validation	[38]
Cosmetic formulations	Peel extracts; pumpkin seed oil and pumpkin polysaccharides incorporated into a multicomponent emulsion	Chemical antioxidant and tyrosinase assays, spectrophotometrically estimated in vitro SPF, and keratinocyte viability; compositional rationale for emollient use	Early laboratory and compositional evidence	No demonstrated clinical sunscreen or dermatological efficacy; irritation, sensitization, phototoxicity, effective dose and finished-product stability remain unestablished	[36]
Extract-mediated silver nanoparticles	Fruit-fiber or peel extracts used as reducing and capping agents	Formation and physicochemical characterization of silver nanoparticles, with microbial growth inhibition in vitro	Exploratory synthesis and in vitro research	Biological activity and toxicity must be attributed to the complete nanoparticle system, not to pumpkin extract alone; cytotoxicity, dose–response relationships, migration, environmental fate, batch reproducibility, and regulatory status require evaluation	[124]

Abbreviations: HPH, high-pressure homogenization; PEF, pulsed electric field; SPF, sun protection factor. A log reduction denotes a base-10 reduction in viable microbial count. Evidence level/readiness indicates the highest level directly demonstrated for the specified matrix and does not imply clinical efficacy, regulatory approval, or commercial readiness.

## Data Availability

No new data were created or analyzed in this study. Data sharing is not applicable to this article.

## Abbreviations

The following abbreviations are used in this manuscript:

FAOSTAT	Food and Agriculture Organization Corporate Statistical Database
PRISMA	Preferred Reporting Items for Systematic Reviews and Meta-Analyses
HPLC-DAD	High-performance liquid chromatography with diode-array detection
LC-MS	Liquid chromatography–mass spectrometry
GC-FID	Gas chromatography with flame-ionization detection
HPLC	High-performance liquid chromatography
GC	Gas chromatography
SC–CO_2_	Supercritical carbon dioxide
SFE/SFE–CO_2_	Supercritical fluid extraction/supercritical fluid extraction using carbon dioxide
DES	Deep eutectic solvent
NADES	Natural deep eutectic solvent
EAAE	Enzyme-assisted aqueous extraction
UAE	Ultrasound-assisted extraction
MAE	Microwave-assisted extraction
HPH	High-pressure homogenization
PEF	Pulsed electric field
LAB	Lactic acid bacteria
CFU	Colony-forming unit
DPPH	2,2-Diphenyl-1-picrylhydrazyl
FRAP	Ferric reducing antioxidant power
HPTP	High-pressure-assisted thermal processing
PPO	Polyphenol oxidase
HbA1c	Glycated hemoglobin (hemoglobin A1c)
ABTS	2,2′-Azino-bis(3-ethylbenzothiazoline-6-sulfonic acid)
TNF-α	Tumor necrosis factor alpha
LUTS	Lower urinary tract symptoms
GRANU	German Research Activities on Natural Urologicals
BPH	Benign prostatic hyperplasia
ALT	Alanine aminotransferase
AST	Aspartate aminotransferase
iAUC	Incremental area under the curve
HDL	High-density lipoprotein
NAFLD	Nonalcoholic fatty liver disease
IPSS	International Prostate Symptom Score
CI	Confidence interval
SPF	Sun protection factor
